# Digenic impairments of haploinsufficient genes in patients with craniosynostosis

**DOI:** 10.1172/jci.insight.176985

**Published:** 2025-02-24

**Authors:** Jung Woo Yu, Jihoon G. Yoon, Chaerim Han, Shin Hye Noh, Dong Min Shin, Yu-Mi Yang, Yong Oock Kim, Kyu-Won Shim, Min Goo Lee

**Affiliations:** 1 Department of Pharmacology, Graduate School of Medical Science Brain Korea 21 Project,; 2 Department of Pediatric Neurosurgery, Craniofacial Reforming and Reconstruction Clinic,; 3 Department of Laboratory Medicine, Gangnam Severance Hospital, and; 4 Severance Biomedical Science Institute, Yonsei University College of Medicine, Seoul, Republic of Korea.; 5 Department of Oral Biology, Yonsei University College of Dentistry, Seoul, Republic of Korea.; 6 Department of Plastic and Reconstructive Surgery, Institute for Human Tissue Restoration, Yonsei University College of Medicine, Seoul, Republic of Korea.

**Keywords:** Bone biology, Genetics, Bioinformatics, Bone disease, Genetic diseases

## Abstract

Craniosynostosis (CRS) is characterized by the development of abnormal cranial suture ossification and premature fusion. Despite the identification of several associated genetic disorders, the genetic determinants of CRS remain poorly understood. In this study, we conducted integrative analyses on 225 exomes, comprising 121 CRS probands and 104 parental exomes (52 trios). These analyses encompassed de novo and pathogenic variants, and digenic combinations within haploinsufficient genes harboring rare variants. Our analysis unveils a shared molecular network between genes associated with CRS and those linked to skeletal and neurodevelopmental disorders, with a notable enrichment of deleterious variants within haploinsufficient genes. Additionally, we identified a unique digenic pair (*IL6ST* and *TRPS1*) within haploinsufficient genes that was present in 2 patients with nonsyndromic CRS but absent in parents or 1,048 population controls. In vitro experiments provided evidence that the identified missense variants were hypomorphs, and accelerated bone mineralization could result from the additive effects of diminished *IL6ST* and *TRPS1* activities in osteoblasts. Overall, our study underscores the important role of rare variations in haploinsufficient genes and suggests that in a subset of undiagnosed patients, the CRS phenotype may arise from multiple genetic variations.

## Abstract 2

This study underscores the significant role of rare variations in haploinsufficient genes and suggests that the CRS phenotype may arise from multiple genetic variations.

## Graphical abstract

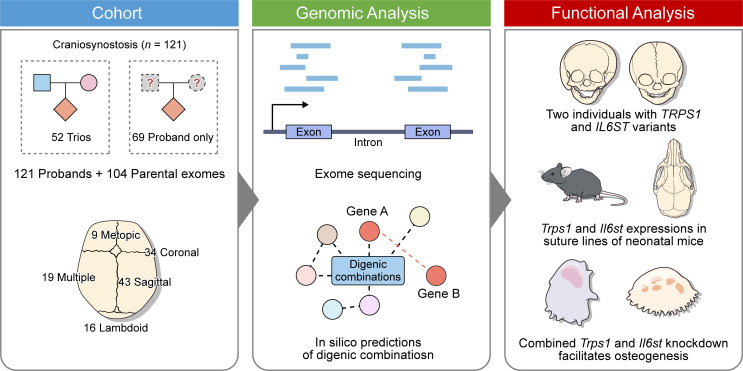

## Introduction

The development of the human brain is intricately tied to the ossification process of cranial sutures. Premature fusion of these sutures gives rise to craniosynostosis (CRS), a pathological condition that not only alters the shape of the skull bone but also affects brain development, potentially leading to neurodevelopmental disorders (NDDs) and intellectual disabilities (1). CRS is widely acknowledged to have a substantial genetic component, and while approximately 100 genes have been identified as monogenic causes of CRS (2, 3), most of the genetic defects have been identified in syndromic cases. Previous studies have highlighted the burden of de novo variants within the WNT, BMP, and Ras/ERK signaling pathways for nonsyndromic cases and in chromatin modification, transcriptional regulation, and retinoic acid signaling pathways for syndromic cases (4, 5). However, the genetic architecture of CRS, in particular that of nonsyndromic CRS, remains poorly understood. Moreover, the identified variants have occasionally exhibited weak phenotypic associations with incomplete penetrance.

Despite advancements in sequencing technologies that have facilitated genomic exploration, identifying the causative genes responsible for human traits within the context of Mendelian inheritance remains a challenge. In our recent investigation using the target sequencing panel for 34 CRS-related genes, we observed that individuals with rare or structural variations in causative genes accounted for up to 30% of CRS patients in a Korean cohort (6), including incomplete penetrance in the haploinsufficiency genes that are associated with autosomal dominant disorders, such as *ERF* and *TCF12* (7, 8). Recent studies have shed light on the oligogenic nature of various disorders, including congenital heart defects, microcephaly, and brain malformation (9–12). In CRS, a previous study suggested a 2-locus model for nonsyndromic midline CRS involving interaction between *SMAD6* rare variants and a common *BMP2* allele (13); however, this finding has been contradicted by another study (14). Additionally, a recent study showed that the digenic combinations of *IGF1* and *RUNX2* may contribute to the pathogenesis of CRS in mouse models (15). Nevertheless, no comprehensive investigation to our knowledge has explored the digenic or oligogenic model for human CRS on a genome-wide scale.

Identification of causative digenic or oligogenic pairs from the vast number of potential gene-gene interactions remains challenging, despite the development of various computational tools to identify oligogenic combinations (16, 17). Here, we hypothesized that CRS could be induced by the additive effect of 2 or more rare variants in haploinsufficient genes. To address this, we focused on digenic combinations within haploinsufficient genes defined by a probability of loss-of-function intolerance (pLI) score greater than 0.9, calculated based on a large human population (18). Our approach is supported by previous findings demonstrating the dosage effect of CRS-related genes in both humans (7, 8) and mice (15), as well as the notable genetic effects resulting from de novo or transmitted rare variants in the constrained genes (19). Initially, we analyzed de novo and pathogenic variants in 121 CRS probands. Subsequently, we examined the 52 trio exomes along with parental and population controls, identifying patient-specific digenic combinations in the haploinsufficient genes. Further analysis of 69 proband-only exomes revealed an additional case with rare variants in the *TRPS1* and *IL6ST* genes, previously undiagnosed. Finally, we conducted in vitro experiments on this gene pair to examine their functional activities and effects on bone ossification.

## Results

### Molecular profiling of CRS patients using exome sequencing.

**Figure 1:**
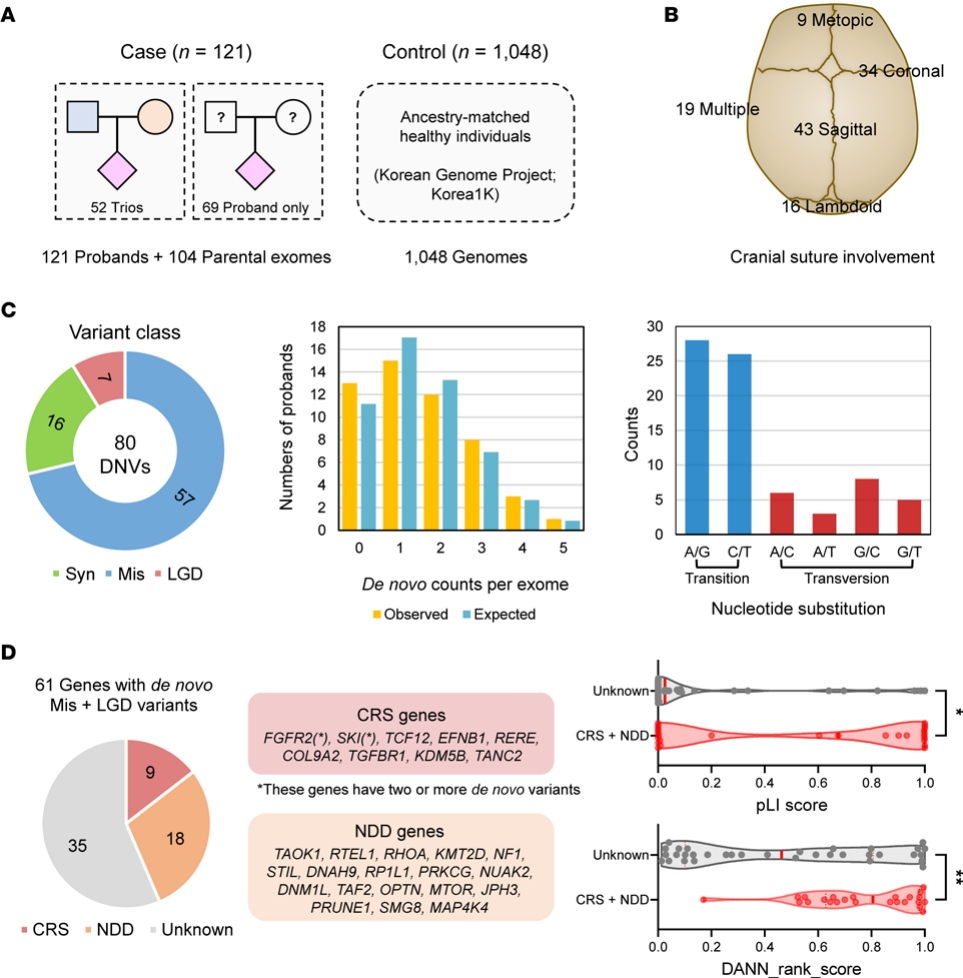
Study cohort and characterization of de novo variants identified in 52 trios. (**A**) Overview of the CRS cohort and the Korean population control (Korea1K). Samples from 121 CRS probands — including 52 trio and 69 proband-only samples — underwent exome sequencing. A population control dataset comprising whole-genome sequencing data from 1,048 unaffected Korean individuals was utilized. (**B**) Distribution and classification of affected cranial sutures in the study cohort. (**C**) Distribution and classification of 80 de novo variants identified in the 52 trios. The observed de novo variant counts per exome did not substantially deviate from the expected Poisson distribution. Mis, missense; Syn, synonymous. (**D**) Analysis revealed the presence of LGD or missense variants in 27 genes associated with CRS or NDDs. These genes exhibited a higher degree of haploinsufficiency and deleteriousness, as indicated by higher pLI scores and DANN rank score parameters. *P < 0.05, **P < 0.01 by Mann-Whitney *U* test.

This study included a total of 121 unrelated Korean patients with CRS, comprising 28 newly enrolled individuals (23.1%) and 93 (76.9%) for whom definitive causative genes had not been identified in a previous targeted gene panel sequencing (6) (Supplemental Table 1; supplemental material available online with this article; https://doi.org/10.1172/jci.insight.176985DS1). The male sex was more prevalent, accounting for 61.2% of all cases; and nonsyndromic cases (*n* = 77, 63.6%) were more common than syndromic ones (*n* = 44, 36.4%). We performed exome sequencing on 52 trios (patient and both parents) and 69 proband-only samples (patients without available parental samples; Figure 1A). For ancestry-matched controls, we utilized genome sequencing data from 1,048 healthy Korean individuals obtained from the Korean Genome Project (20). The majority of CRS cases (84.3% [*n* = 102]) exhibited involvement of a single cranial suture, specifically with 43 sagittal, 34 coronal, 16 lambdoid, and 9 metopic sutures affected (Figure 1B). None of the patients had a family history of CRS. The relatedness and ancestry prediction analyses confirmed that all patients enrolled in this study were unrelated and of East Asian origin (Supplemental Figure 1).

First, we focused on analyzing de novo variants in the 52 trios. A total of 80 de novo variants in protein-coding regions or nearby junctions were identified (Figure 1C). These variants consisted of 76 single nucleotide variants (SNVs) and 4 small indels (Supplemental Table 2). The distribution of de novo variant counts across the trio samples followed an expected Poisson distribution, indicating no significant deviation from the expected pattern. The transition-to-transversion (Ti/Tv) ratio for de novo SNVs was calculated as 2.45. We further explored the burden of de novo variants in the CRS probands and observed a notable enrichment of protein-altering variants, specifically a 1.65-fold enrichment in missense variants (*P* = 4.1×10^–4^, Supplemental Figure 2A). No significant difference was observed in the number of de novo variants according to parental age in our study (Supplemental Figure 2B). In this analysis, we identified 64 protein-altering variants (missense and likely gene-disrupting [LGD]) in 61 genes, which were further classified into 3 categories: CRS-, NDD-, and unknown-associated genes. Pathway analysis of these genes highlighted their involvement in biological pathways related to neurogenesis, neuron differentiation, and cranial suture abnormalities (Supplemental Figure 3, A and B). Among these genes, 26 were previously associated with CRS or NDDs. Furthermore, when comparing the characteristics of genes in the CRS/NDD and the unknown categories, we found that the CRS/NDD genes exhibited higher pLI scores (median 0.64 vs. 0.02, *P* = 0.020) and had higher deleterious in silico predictions (median deleterious annotation of genetic variants using neural networks [DANN] rank score 0.74 vs. 0.46, *P* = 0.007) than the unknown genes (Figure 1D). These results suggest that genes contributing to CRS phenotypes are more likely to harbor deleterious variants in haploinsufficient genes.

Subsequently, we investigated rare variants potentially associated with Mendelian disorders. Our analysis unveiled 16 de novo pathogenic (P) or likely pathogenic (LP) variants in 15 probands, which were linked to autosomal or X-linked dominant disorders in 28.8% of the 52 trio-sequenced samples (Table 1). CRS diagnosis in these probands was confirmed through 3D reconstructions obtained from skull CT scans, and de novo variants were validated by Sanger sequencing (Supplemental Figure 4). Overall, we identified 41 probands (33.9%) with P/LP variants within 33 genes (Supplemental Table 3). While some variants were found in well-known CRS genes such as *FGFR2*, *TCF12*, *EFNB1*, and *SKI*, we also identified P/LP variants in genes associated with NDDs and skeletal disorders, such as *NF1*, *RERE*, *COL9A2*, *TAOK1*, *RTEL1*, *TGFBR1*, *MTOR*, *KMT2D*, and *TANC2*. Pathway analysis of the 33 genes with P/LP variants highlighted their involvement in biological pathways related to neuronal or osteoblastic differentiation and bone ossification (Supplemental Figure 3, C and D). These findings highlight the close relationship between CRS and the molecular networks underlying NDDs and skeletal disorders, implying that CRS may manifest as a phenocopy or a phenotypic expansion of one or both of these types of disorders.

Furthermore, we identified 3 probands with multilocus pathogenic variations (MPVs) (21, 22) involving the *SMAD6*, *TCF12*, and *FBN1* genes (Table 2). Interestingly, variants in the *SMAD6* and *TCF12* genes often exhibit incomplete penetrance. Therefore, the present findings support the notion that the CRS can arise as phenotypic expansion from multiple genetic hits. Additional pathogenic variants would increase the penetrance of CRS, while their absence in many cases may account for reduced penetrance (23).

### Identification of digenic impairments of haploinsufficient genes in CRS patients.

**Figure 2:**
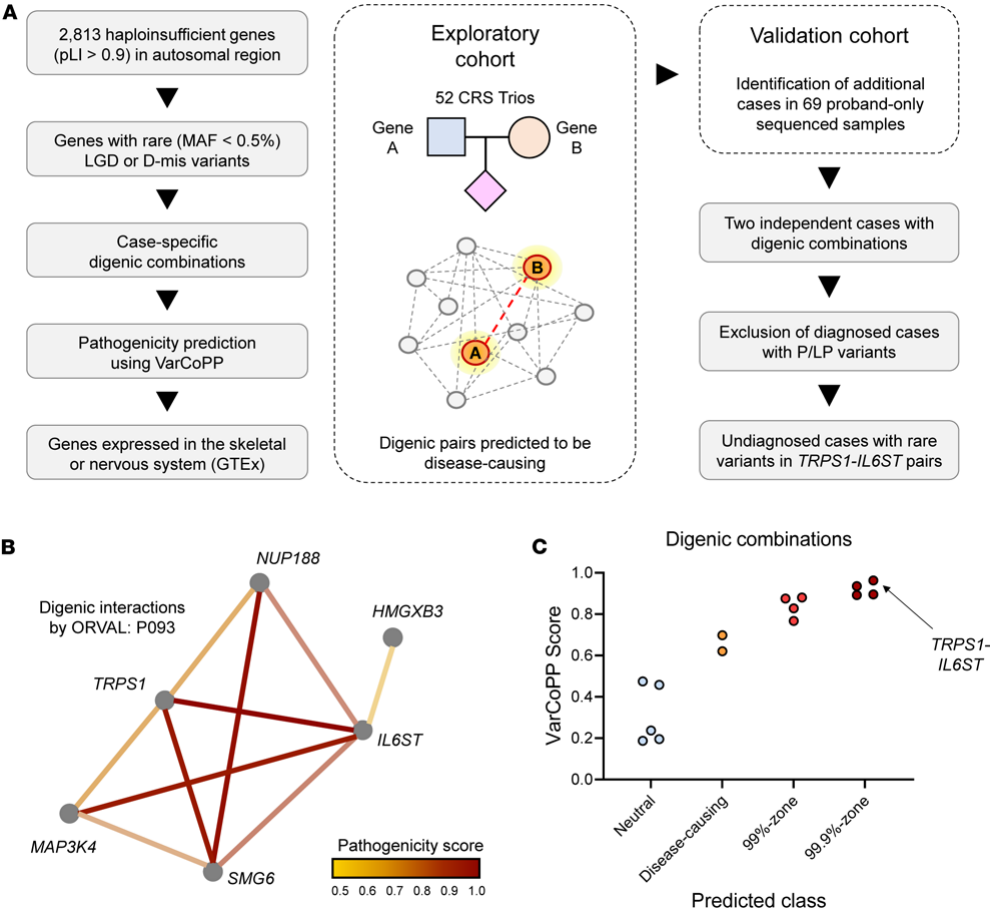
Identification of digenic combinations in haploinsufficient genes associated with CRS. (**A**) Investigation of rare digenic combinations in haploinsufficient genes (pLI > 0.9) associated with CRS. Rare deleterious variants, including LGD or D-mis) variants, were screened in 52 CRS trios. Digenic combinations observed in unaffected individuals were filtered using unaffected parents and population controls, with only those present exclusively in affected probands retained. The pathogenicity of case-specific digenic combinations was predicted using VarCoPP. Further analysis focused on digenic pairs falling within the 99.9% confidence zone of disease-causing variants and involved additional sequencing of 69 CRS probands. Through this approach, the *TRPS1* and *IL6ST* gene pair was identified, observed in 2 independent undiagnosed cases. (**B**) Illustration of digenic interactions predicted by ORVAL in patient P093. Rare variants were filtered, and their pathogenicity was predicted based on gene-gene interaction networks, following the aforementioned process. (**C**) Prioritization of digenic combinations based on VarCoPP pathogenicity scores classified in the 99.9% zone. The validation cohort of 69 proband-only samples was then evaluated to identify additional undiagnosed cases harboring these digenic combinations.

We observed a high proportion of haploinsufficient genes among the known CRS/NDD genes (Figure 1D). Also, the well-established dosage effect observed in the *ERF* and *TCF12* genes (7, 8) on the CRS phenotype led us to hypothesize that haploinsufficient genes might play a crucial role in the development of CRS (24). Consequently, we conducted exome-wide screening for possible digenic combinations in CRS cases (Figure 2A). We focused on 2,813 autosomal haploinsufficient genes (pLI > 0.9) and rare functionally deleterious (LGD) or deleterious missense (D-mis) variants. We conducted this analysis using an exploratory cohort of 52 trios, their 104 parents, and a control dataset of 1,048 healthy individuals from the Korean population. We identified CRS-specific digenic pairs by excluding combinations found in parents or population controls. The pathogenicity of these pairs was evaluated using ORVAL (16), with only those classified in the “99.9%-zone disease-causing” category retained (Figure 2, B and C). Further analysis of additional CRS proband-only samples led to the identification of 6 digenic pairs present in 2 independent cases (Supplemental Table 4).

Notably, all identified gene variants from the 6 pairs were D-mis variants of genes expressed in the brain and skeletal system. However, in 3 gene pairs, P/LP variants related to CRS genes (*FGFR2*, *TCF12*, and *ERF*) were also identified in 1 of the 2 patients in each gene pair group (Supplemental Table 4). While this may, in part, explain the low penetrance of some CRS genes (*TCF12* and *ERF*), we excluded them from further analysis to focus on the digenic pairs with pure multiple cases. Additionally, 2 digenic pairs with additional variations in NDD genes (*KMT2D* and *RERE*) in 1 of the 2 patients were deprioritized for similar reasons. As a result, we focused on cases harboring D-mis variants in the *TRPS1* and *IL6ST* genes.

### Digenic impairments of IL6ST and TRPS1 in nonsyndromic CRS.

**Figure 3:**
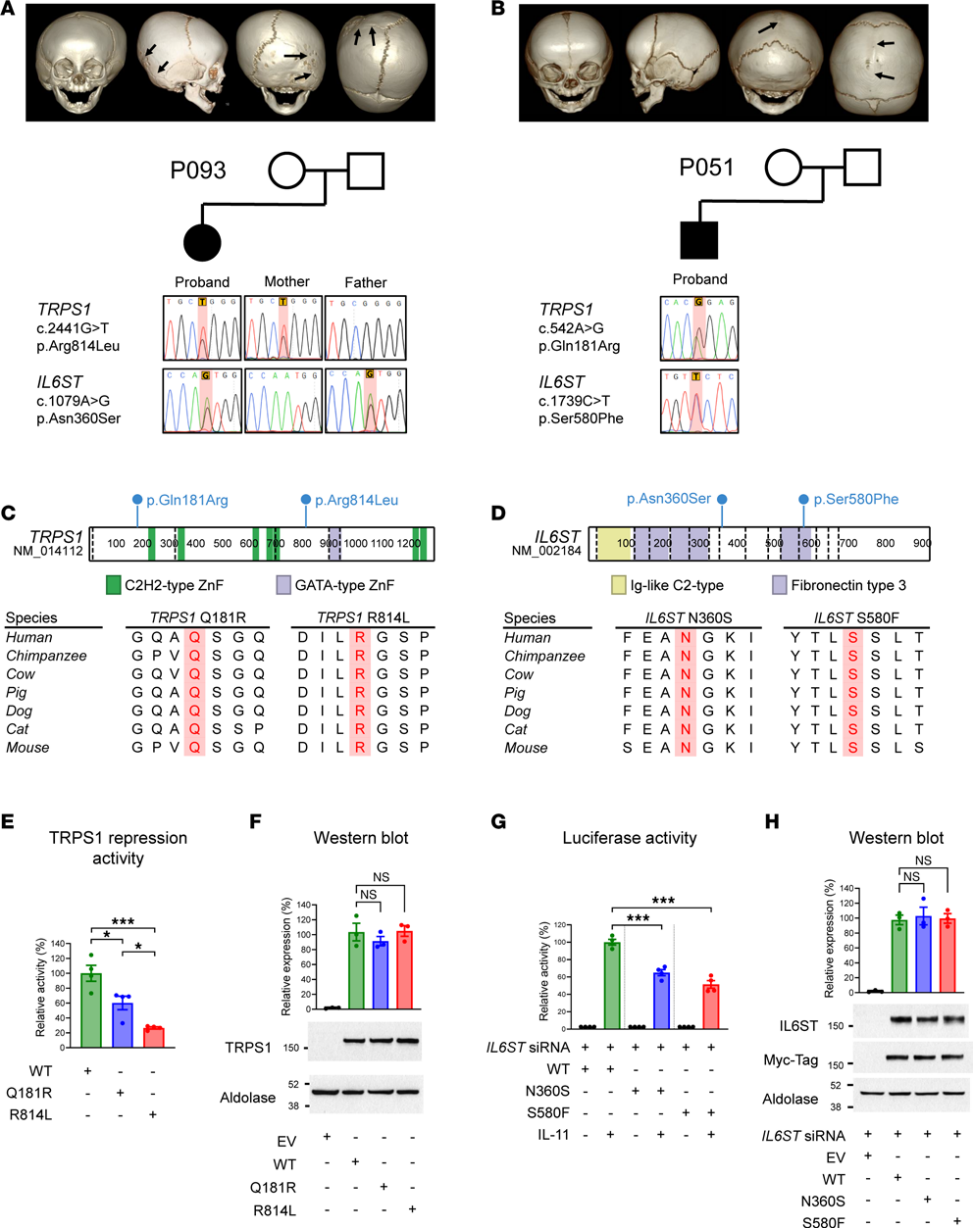
Characterization of CRS patients with digenic impairments in *TRPS1* and *IL6ST*. (**A** and **B**) Pedigree and genotype information of the 2 individuals with rare variants in *TRPS1* and *IL6ST*. 3D reconstruction images of skull computed tomography reveal premature fusions of lambdoidal and sagittal sutures (indicated by black arrows) in P093 and P051, respectively. (**C** and **D**) Schematic representation of the variants identified in *TRPS1* and *IL6ST*. The positions of all variants are indicated, and they are shown to be located in highly conserved sites across mammalian species. (**E**) TRPS1 functional activity was measured using the osteoblast-specific *cis*-acting element (OSE2) reporter assay in HepG2 cells, which minimally express endogenous *TRPS1*. Cells were cotransfected with plasmids for *RUNX2* to activate OSE2 reporter transcriptional activity. Subsequently, TRPS1-mediated transcriptional repression activity was measured using the dual luciferase assay in cells transfected with WT or variant *TRPS1* plasmids. The Q181R and R814L variants showed lower TRPS1 activity than WT. EV, empty vector. (**F**) Immunoblot analyses show comparable protein expression levels among TRPS1 WT and variant proteins. (**G**) IL6ST functional activity was measured using the IL6 SIE/STAT3 dual luciferase reporter assays with IL-11 stimulation in HEK293 cells. To eliminate the potential effects of endogenous IL6ST, we treated cells with siRNA against the 3′-UTR of *IL6ST* 6 hours before plasmid transfection. Cells were then transfected with plasmids for dual luciferase assay and plasmids for the expression of IL-11 receptor and IL6STs (WT, N360S, and S580F). The N360S and S580F variants showed lower IL6ST functional activity than WT. (**H**) Immunoblot analyses show comparable protein expression levels among WT and variant types of IL6ST. n.s., not significant. *P < 0.05, ***P < 0.001.

We identified 2 unrelated patients (P051 and P093; Supplemental Figure 5) with nonsyndromic CRS who carried heterozygous D-mis variants in *TRPS1* (NM_014112) and *IL6ST* (NM_002184). The affected cranial sutures were the sagittal and right lambdoidal lines in patients P051 and P093, respectively (Figure 3, A and B). The identified variants were *TRPS1* c.2441G>T, p.Arg814Leu and *IL6ST* c.1079A>G, p.Asn360Ser for P093; and *TRPS1* c.542A>G, p.Gln181Arg and *IL6ST* c.1739C>T, p.Ser580Phe for P051. These variants were found at highly conserved sites across mammalian species (Figure 3, C and D). The structural prediction software AlphaFold predicted with high confidence that the N360S and S580F variants in the *IL6ST* gene (encoding GP130) were situated in the fibronectin type III domains of extracellular regions (Supplemental Figure 6A). In contrast, the *TRPS1* Q181R and R814L variants were located in poorly predicted regions (Supplemental Figure 6B).

The *TRPS1* and *IL6ST* genes are associated with trichorhinophalangeal syndrome (OMIM #190350) and Stuve-Wiedemann syndrome (OMIM #619751) in autosomal dominant and recessive modes, respectively. However, neither patient did exhibited clinical features of trichorhinophalangeal syndrome. Functional characterization of *TRPS1* WT, Q181R, and R814L variants using the dual-luciferase reporter assay and HepG2 cells (Supplemental Figure 7) indicated that these variants were hypomorphs. Specifically, the *TRPS1* Q181R and R814L variants exhibited reduced *TRPS1*-mediated transcriptional repressor activity on the OSE2 reporter activation, with WT levels of 60% and 26.7%, respectively, of those of WT (Figure 3E). Immunoblot analyses revealed that protein expression levels were not altered within *TRPS1* variants (Figure 3F).

Similarly, the functional activity of *IL6ST* variants was analyzed using the IL6 sis-inducible element (SIE/STAT3) dual-luciferase reporter assay upon IL-11 stimulation with the exogenous expression of WT and *IL6ST* variants in HEK293 cells (Figure 3G). The cells were pretreated with an siRNA against the 3′-UTR of *IL6ST* to eliminate confounding effects incurred from the endogenous IL6ST (Supplemental Figure 7). The IL6ST activity measurements using the reporter assays revealed that the *IL6ST* N360S and S580F variants exhibited decreased activity, with levels that were 65% and 48%, respectively, of those of WT (Figure 3G). Control immunoblot analyses showed that the *IL6ST* variants did not affect protein abundance (Figure 3H). Collectively, our experimental findings indicate that the *IL6ST* and *TRPS1* variants identified in the patients were hypomorphs, resulting in reduced functional activity, while each genetic variation was not sufficient to cause a pathogenic effect.

### Accelerated bone mineralization in osteoblasts induced by Il6st and Trps1 downregulation.

**Figure 4:**
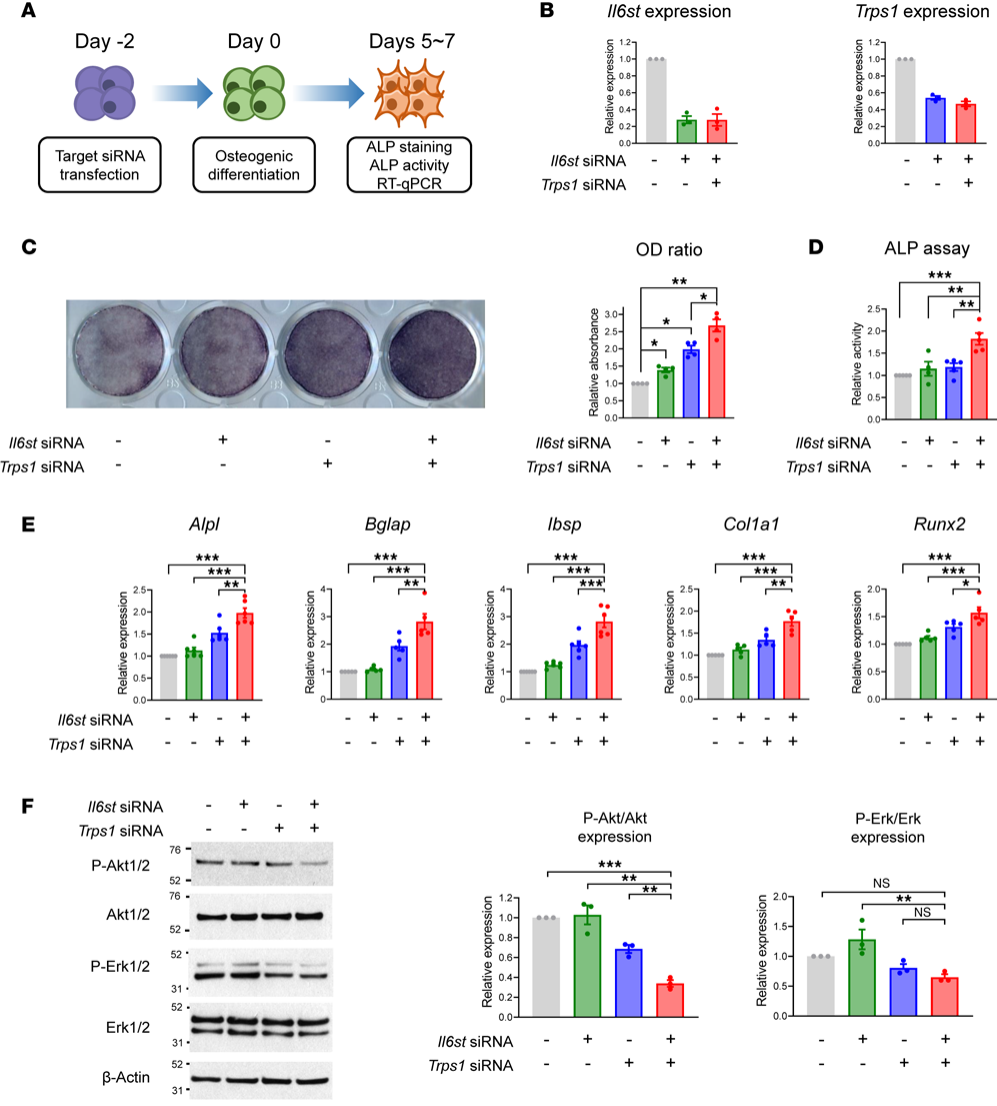
Additive effects of *Trps1* and *Il6st* double knockdown on bone mineralization in osteoblast. (**A**) Schematic representation of the osteoblast differentiation process in mouse calvarial cells (MC3T3-E1). *Il6st* and *Trps1* siRNA treatments were performed 48 hours before induction of differentiation. The osteogenic medium was replenished every 3 days, and differentiation was evaluated on days 5–7 after osteogenic induction. (**B**) Measurement of *Il6st* and *Trps1* expression levels using qRT-PCR after 48 hours of siRNA transfection (*Il6st*: 25 nM, *Trps1*: 25 nM) with Lipofectamine RNAiMAX (*n* = 3, biologically independent samples). (**C**) ALP staining was performed on day 5 after the induction of differentiation. The double-siRNA-treated groups exhibited the most prominent staining, as measured by the highest optical-density (OD) ratio. (**D**) ALP activity was measured using ELISA on day 5 after the induction of differentiation. The double-siRNA-treated groups exhibited the most prominent ALP activity, indicating the highest level of osteoblast differentiation. (**E**) Measurement of osteogenic markers (*Alpl*, *Bglap*, *Ibsp*, *Col1a1*, and *Runx2* mRNA) by qRT-PCR on day 5 after osteogenic induction (*n* = 5 or 6, biologically independent samples). (**F**) Representative Western blot images representing the protein abundance of phosphorylated Akt1/2 (P-Akt1/2) and phosphorylated Erk1/2 (P-Erk1/2) in the MC3T3E1 cell line after the 6-hour differentiation period. Densitometry-based quantification of the Western blot results, presenting relative protein expression levels as mean ± SD (*n* = 3, biological replicates). Remarkably, double-knockdown groups treated with *Il6st* and *Trps1* siRNA exhibited synergistic effects on phospho-*Akt* levels, consistent with the observed trends in osteogenic markers. *P < 0.05, **P < 0.01, ***P < 0.001.

To investigate the effects of *IL6ST* and *TRPS1* hypomorphs on intramembranous ossification, simultaneous knockdowns of *Il6st* and *Trps1* were conducted in the MC3T3E1 murine calvarial cells using combinations of gene-specific siRNAs (Figure 4A). The knockdown efficiency of each gene was measured via quantitative real-time PCR (qRT-PCR) analysis, 48 hours after the siRNA transfection. As shown in Figure 4B, a 50%–70% reduction in mRNA levels in the target gene was achieved via transfections with appropriate concentrations of each siRNA. Combinations of both siRNAs did not greatly affect the mRNA levels of each gene, indicating no significant interference between the siRNAs used (Figure 4B). Analyses of alkaline phosphatase (ALP) staining results revealed that knockdowns of *Il6st* resulted in no significant change, whereas *Trps1* exerted a partial increase in bone mineralization. Notably, the combined knockdown of *Il6st* and *Trps1* resulted in a synergistic effect, showing the highest level of bone mineralization in ALP staining (Figure 4C). The synergistic effect of *Il6st* and *Trps1* double knockdown was further confirmed using an ELISA-based ALP activity assay (Figure 4D). Furthermore, mRNA expression levels of genes associated with osteogenic differentiation, including *Alpl*, *Bglap*, *Ibsp*, *Col1a1*, and *Runx2*, showed an additive or potentiation effect of *Il6st* and *Trps1* double knockdown on osteoblastogenesis in the MC3T3E1 cells (Figure 4E). In an immunoblot assay identifying the signaling pathways responsible for bone mineralization, the phosphorylated forms of AKT were prominently reduced in the cells with the double gene knockdown (Figure 4F). In aggregate, these results imply that simultaneous reductions in IL6ST and TRPS1 activity can accelerate bone mineralization, potentially contributing to the development of CRS, as observed in our patients.

**Figure 5:**
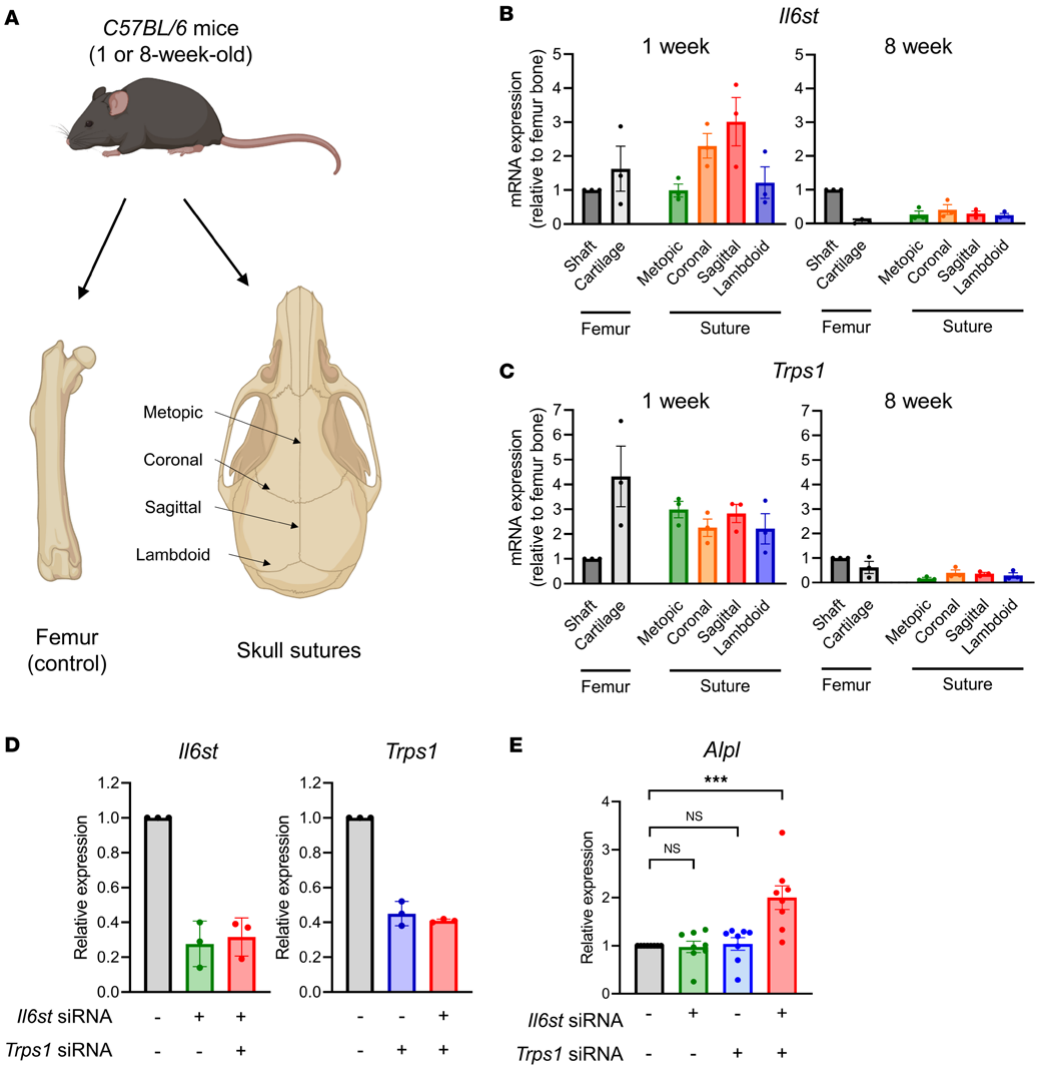
Expression of *Il6st* and *Trps1* in mouse cranial sutures and the effects of double knockdown in primary osteoblast differentiation. (**A**) Schematic illustration of mouse tissue samplings, including femur and calvarial sutures, with indications of metopic, coronal, sagittal, and lambdoidal sutures. (**B** and **C**) Relative expression levels of *Il6st* and *Trps1* in 1-week-old and 8-week-old mice. Bone marrow cells from the femur shaft were used as controls. During the neonatal period (1 week old), *Il6st* and *Trps1* are abundantly expressed in cranial sutures. In contrast, their expression levels are markedly reduced upon suture closure at 8 weeks of age (*n* = 3, biological replicates). (**D**) Effective knockdown of *Il6st* and *Trps1* mRNA levels in primary cultures of mouse osteoblasts following transfection with siRNAs targeting each gene (*Il6st*: 25 nM, *Trps1*: 50 nM). (**E**) Expression levels of the osteogenic marker gene *Alpl* in primary cultures of mouse osteoblasts (*n* = 8, biological replicates). Data are presented as mean ± SEM. ***P < 0.001, as determined by 1-way ANOVA followed by Tukey’s multiple-comparison test.

We then conducted similar experiments using primary cultures of mouse osteoblasts obtained from cranial sutures (Figure 5A). Notably, *Il6st* and *Trps1* mRNAs were abundantly expressed in the cranial sutures of neonatal mice (1 week old), whereas their expression levels were markedly lower in sutures that had closed (8 weeks old) (Figure 5, B and C). Transfection with *Il6st* and *Trps1* siRNAs resulted in 60%–70% reductions in the respective mRNA levels (Figure 5D). Interestingly, simultaneous knockdown of *Il6st* and *Trps1* significantly increased expression of the osteogenic marker *Alpl* in primary cultures of mouse osteoblasts, whereas knockdown of each gene individually did not lead to significant changes (Figure 5E). Again, this indicates that the combined downregulation of *Il6st* and *Trps1* promotes osteogenesis.

### Trps1 downregulation suppresses osteoclast differentiation.

**Figure 6:**
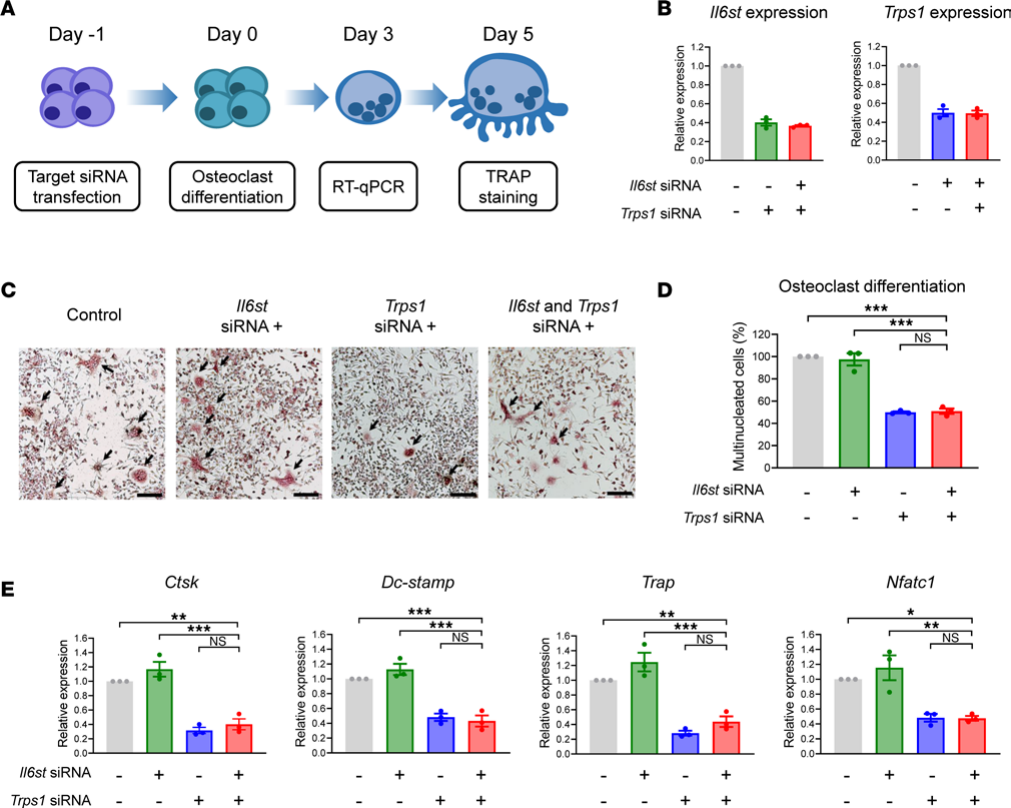
Downregulation of *Trps1* suppresses osteoclastogenesis. (**A**) Schematic of the osteoclast differentiation process in mouse macrophages (RAW 264.7). The cells were cultured and transfected with siRNA 24 hours before differentiation, which was induced by the addition of RANKL. qRT-PCR and TRAP staining were performed on day 3 and day 5, respectively. (**B**) Measurement of *Il6st* and *Trps1* expression levels using qRT-PCR after 48 hours of siRNA transfection (*Il6st*: 12.5 nM, *Trps1*: 25 nM) using TransIT-LT1 Transfection Reagent (*n* = 3, biologically independent samples). (**C**) Representative TRAP staining images on day 5 after initiation of differentiation (Scale bars: 100 μm). Osteoclasts (multinucleated cells) are indicated by black arrows. (**D**) Osteoclast differentiation was quantified by measuring multinucleated cells, normalized to 100% based on the control (scrambled group). (**E**) Expression analysis of osteoclast differentiation marker genes (*Ctsk*, *Dc-stamp*, *Trap*, and *Nfatc1*) using qRT-PCR on day 3 (*n* = 3, biologically independent samples). Expression levels of osteoclast differentiation markers further confirmed that the combined knockdown of *Trps1* and *Il6st* does not induce an additive effect on osteoclastogenesis. *P < 0.05, **P < 0.01, ***P < 0.001.

Considering that bone homeostasis is achieved via a balanced regulation of osteoblasts and osteoclasts, we also assessed the effect of *Trps1* and *Il6st* downregulation on osteoclastogenesis using RAW 264.7 cells (Figure 6A). We used qRT-PCR analyses to determine knockdown efficiency (48 hours after siRNA transfection) and found that a 50%–60% reduction in *Trps1* and *Il6st* mRNA expression was achieved via treatment with appropriate concentrations of gene-specific RNAs in RAW 264.7 cells (Figure 6B). To assess osteoclastogenesis and osteoclast differentiation, we analyzed for the presence of differentiated multinucleated giant cells using tartrate-resistant acid phosphatase (TRAP) staining. Interestingly, cells treated with siRNAs against *Trps1* displayed reduced osteoclast differentiation, while those treated with siRNAs against *Il6st* did not exhibit discernible changes. Furthermore, no additive effects were observed in cells treated with combinations of both siRNAs (Figure 6, C and D, and Supplemental Figure 8). Similar findings were also identified in the quantification of mRNA expression levels of osteoclast differentiation markers, such as *Ctsk*, *Dcstamp*, *Trap*, and *Nfatc1* (Figure 6E). Collectively, these results suggest that the downregulation of *Trps1* alone hinders osteoclastogenesis, while there are no additional effects when *Il6st* is simultaneously reduced.

## Discussion

Our understanding of the molecular mechanisms of CRS remains limited, particularly in nonsyndromic cases, where diagnostic success rates have been lower than those in syndromic cases (1). Despite the identification of more than 100 genes linked to CRS, their clinical interpretation for molecular diagnosis has been challenging due to factors such as incomplete penetrance and weak associations (2, 3). To address these knowledge gaps, our study aimed to investigate the genetic architecture of CRS by utilizing exome sequencing in a cohort of 121 CRS probands. Specifically, we explored the potential role of digenic rare variants involving haploinsufficient genes in undiagnosed cases. Our results underscore the importance of de novo variants and burdens of rare variants in haploinsufficient genes as major genetic drivers of CRS. Our analyses revealed that genes associated with CRS shared a molecular network with genes linked to NDD and skeletal disorders (Supplemental Figure 3). Notably, we observed a high burden of de novo missense variants in CRS and NDD genes, predominantly occurring in haploinsufficient genes.

By focusing on digenic combinations within haploinsufficient genes, we were able to identify 6 candidate cases that may be overlooked in a monogenic framework. Among these gene pairs, both harbored D-mis variants in the *TRPS1* and *IL6ST* genes, and disease remained undiagnosed, with no P/LP variants associated with CRS or NDDs (Supplemental Tables 3 and 4). Therefore, we evaluated the functional effects of these variants on the mechanisms of CRS. Notably, the present experimental results indicate that simultaneous *Trps1* and *Il6st* impairments lead to accelerated bone mineralization in MC3T3E1 calvarial osteoblasts, closely resembling CRS development. This suggests that the interaction between these 2 genes may play a pivotal role in cranial suture development and contribute to the pathogenesis of CRS.

The role of *TRPS1* as a repressor of bone formation and its association with bone ossification are well established. Previous studies have demonstrated that knockdown of TRPS1 leads to accelerated osteoblast differentiation (25). Our *TRPS1* variants were not located in the mutation hotspots causing trichorhinophalangeal syndrome, such as the GATA-type Zn finger domain (894–952 residues; Figure 3C). The results from the OSE2 reporter assay indicated that the *TRPS1* Q181L and R814 variants were hypomorphs in terms of inhibiting the RUNX2-mediated transcriptional activation. The R814L variant had lower activity compared with the Q181L variant, with a decrease of 73% versus 40% (Figure 3E). The lower activity of the R814L variant may be attributed to its location being close to the domains known to interact with *GLI3*, which play an important role in the downstream signaling of TRPS1 (Figure 3C) (26). *GLI3* is recognized as a repressor of osteoblast function, and loss-of-function variants in *GLI3* have been linked to CRS in the mouse (27).

The GP130 cytokine receptor subunit encoded by *IL6ST* is the shared receptor for the IL-6 cytokine family, including IL-6, IL-11, IL-27, leukemia-inhibitory factor (LIF), oncostatin M (OSM), ciliary neurotrophic factor (CNTF), cardiotrophin 1 (CT1), and cardiotrophin-like cytokine (CLC). It has been reported that some biallelic loss-of-function variations in the *IL6ST* gene are associated with CRS (28, 29), while GP130-mediated signaling in general is known to be required for normal osteoblastic function. The *IL6ST* N360S and S580F variants identified in our patients exhibited low SIE/STAT3-mediated transcriptional activity upon IL-11 stimulation (Figure 3G). Interestingly, partial gene knockdown of *IL6ST* had no effect on osteogenic differentiation, while it showed additive/potentiation effects when combined with *TRPS1* partial knockdown in MC3T3E1 calvarial cells (Figure 4, C–E). Given that the GP130 complex transduces signals from multiple cytokine families at the cell membrane and is responsible for various phenotypes via stimulation and inhibition of multiple signaling pathways, the precise mechanism of IL6ST knockdown appears to be complicated and has yet to be resolved. A potential mechanism would be the upstream and downstream signaling cascades related to AKT activation, of which phosphorylation highly correlated with the osteogenic differentiation induced by *TRPS1* and *IL6ST* gene knockdowns (Figure 4F). Previous studies have demonstrated that inactivation of AKT1 promotes osteoblast differentiation, while reducing osteoclast resorption (30). These findings are consistent with our results and support the potential role of AKT signaling in the development of CRS.

In contrast to the effects in MC3T3E1 cells, only *Trps1* downregulation affected osteoclastogenesis, and the double *Trps1* and *Il6st* knockdown exerted no additive effects in RAW 264.7 cells (Figure 5); this suggests that accelerated osteogenesis could be primarily responsible for the *TRPS1* and *IL6ST* variant–induced CRS. Considering that balanced osteoblast and osteoclast activities determine bone homeostasis, the reduced osteoclastogenesis observed in *TRPS1* hypomorphs may also contribute to the presentation of CRS phenotypes under conditions of marginally increased osteogenesis, influenced by the effects of *TRPS1* and *IL6ST* variants on osteoblasts.

Limitations inherent to our study include the absence of in vivo investigations to assess the biological impact of digenic combinations. Although the results of the present study provide substantial experimental evidence supporting that *TRPS1* and *IL6ST* hypomorphs promote osteogenesis, generating heterozygous mouse models for each gene and cross-breeding them to observe CRS phenocopies would offer more robust evidence to validate our findings. A future study enabling high-throughput investigation of genetic effects resulting from multiple genetic combinations would substantially enhance our understanding of the effects of gene-gene interactions. Furthermore, it is worth noting that our study exclusively examined the effects of combinations involving the *IL6ST* and *TRPS1* genes identified within our cohort. This selection was made due to the presence of P/LP variants in other gene pairs associated with CRS or NDDs (Supplemental Table 4). While these gene pairs merit further evaluation within larger cohorts, it remains a possibility that the genetic variations could contribute to the penetrance or phenotypic variability, as we observed MPVs in 3 patients within our cohort (Table 2) (21, 22).

In the individuals with TRPS1 and IL6ST variants, the suture lines affected with synostosis were both midline (sagittal and lamboidal) (Figure 3, A and B). While coronal synostosis is more frequently associated with monogenic causes (1), the genetic basis of midline synostosis is thought to be more complicated (4). Previous studies have suggested that a polygenic background may contribute to phenotypic variability and penetrance (23). This complexity likely influences the affected suture lines, as seen in our cases of midline synostosis associated with digenic combinations of *TRPS1* and *IL6ST* variants. Furthermore, we observed that substantial variability in *TRPS1* and *IL6ST* expression across developmental stages, anatomical locations, and cell types in mice (Figure 5). These findings highlight the importance of spatial and temporal regulation of these genes in cranial suture biology (31). Future investigations are warranted to better understand the interplay between genetic factors and affected suture lines in humans.

In summary, we have shown that CRS patients have a high burden of deleterious missense variants in haploinsufficient CRS/NDD genes and that interactions among genes carrying rare variants could contribute to the nonsyndromic CRS phenotype. These results highlight the substantial role of rare variants in haploinsufficient genes and expand the molecular framework of CRS to digenic or oligogenic architecture. Future studies with larger cohorts may yield further evidence for the multiple genetic variations contributing to the CRS phenotype and the biological consequences of gene-gene interactions in cranial suture biology.

## Methods

### Sex as a biological variable.

Sex was not considered as a biological variable in this study. Our study included both male and female participants. Similarly, both female and male mice were included and analyzed collectively.

### Participants.

The patient cohort consisted of 121 unrelated Korean pediatric patients who were diagnosed with CRS (Supplemental Table 1). Among 110 patients who underwent targeted gene panel assessment comprising 34 CRS-related genes (6), 93 were selected for exome sequencing, excluding those with definite monogenic causes with complete penetrance, such as *FGFR2*, *FGFR3*, *TWIST1*, and *EFNB1*. Of these 93 cases, 79 remained unresolved after targeted sequencing, while 14 cases presented with incomplete penetrance variants (*TCF12* [*n* = 4], *ERF* [*n* = 3], *ALPL* [*n* = 1], *FBN1* [*n* = 1]) or chromosomal abnormalities with unknown causative genes (1p32-p31 deletion [*n* = 1], 10q26 deletion [*n* = 1], 16p11.2 deletion [*n* = 2], 17p13.3 deletion [*n* = 1]). To supplement the initial patient cohort, an additional 28 new cases were enrolled for initial molecular evaluation between August 2017 and September 2019 at the Craniofacial Reforming and Reconstruction Clinic of Severance Hospital, Seoul. The overall diagnostic workup process has been previously described (6). To serve as population controls, we included a group of 1,048 healthy Korean individuals from the Korean Genome Project (Korea1K) (20) who were matched based on ancestry. Whole-genome sequencing (WGS) data were provided by the Korean Genomics Center at the Ulsan National Institute of Science and Technology (UNIST), and we applied the same quality control steps as those used for exome sequencing, except for filtering with a minimum depth (DP) of 10.

### Whole-exome sequencing and data processing.

Peripheral blood samples were collected from a total of 225 participants, including 52 trio families and 69 proband-only samples. Genomic DNA (gDNA) was extracted from peripheral blood using the QIAamp DNA Mini Kit (QIAGEN) following the manufacturer’s protocol. The concentration and purity of the extracted gDNA were assessed using a Nanodrop spectrophotometer (Thermo Fisher Scientific). A minimum of 500 ng gDNA was used for the preparation of whole-exome sequencing libraries. We employed the xGen Exome Research Panel v1 (Integrated DNA Technologies) for targeted enrichment of exon regions. Library preparation involved shearing the gDNA samples, followed by end-repair, A-tailing, adapter ligation, and PCR amplification. Sequencing was carried out using the NovaSeq 6000 system (Illumina), generating paired-end reads of 150 bp in length. The average depth of coverage was 151×, and the percentage of target regions with coverage above 20× was 96.3%. Germline variant calling was conducted by following the Genome Analysis Tool Kit (GATK; https://gatk.broadinstitute.org/) Best Practices for germline short variant discovery, with modifications from the previous pipeline (6). GATK (v4.1.4), Picard (v2.20.8), and Samtools (v1.9) were utilized, and reads were aligned to the human reference genome of GRCh37/hg19. A joint genotyping and genotype refinement workflow was implemented, followed by the application of a variant hard filter. The variant quality score recalibration (VQSR) tranche was set at ≤99.7 for SNVs and ≤99.0 for indels. Variants were excluded from further analysis if they had genotype quality (GQ) < 20, DP < 20, or a missing genotype rate > 10%, or if they violated the Hardy-Weinberg equilibrium (*P* < 10^–12^) or allelic balance ranges (0.3 < AB < 0.7 for heterozygotes, AB > 0.98 for homozygotes). In addition, we performed quality control for sampling error, sex, relatedness, and ancestry using Peddy (32). ANNOVAR (https://annovar.openbioinformatics.org/) was used for annotation with Ensembl, avsnp150, dbNSFP v4.2a (33), gnomAD v2.1.1 (18), Online Mendelian Inheritance in Man (34), and InterVar (35) databases. All coding or nearby variants were classified into synonymous, missense, in-frame, frameshift, splice site, or nonsense variants. Specifically, we defined LGD variants to include frameshift, nonsense, and splice site (±5 bp) variants that were predicted to be deleterious by dbscSNV (ADA > 0.9) (36). Furthermore, we considered variants with a DANN score above 0.98 to be D-mis (37).

### De novo variant analysis.

De novo variants were analyzed in 52 proband-parent trios. To identify de novo variants, the trio samples were jointly analyzed with pedigree information, and the analysis followed stringent criteria with some modifications (19). In brief, the candidate de novo variants were required to be heterozygous in probands and reference genotypes in both parents and have a population allele frequency of less than 0.1% in the gnomAD v.2.1.1 exome. Only high-confidence sites with GQ ≥ 60, DP ≥ 20, and AB between 0.3 and 0.7 for probands and GQ ≥ 25, DP ≥ 20, and AB < 0.03 for both parents were included. To validate our findings, we performed in silico confirmation of de novo candidates using DeepVariant (38) or Sanger sequencing (Supplemental Figure 4). We only included variants with a “PASS” tag in the FILTER field for further analysis. Finally, we analyzed confirmed de novo variants using the R package “denovolyzeR” (39). The pathogenicity of each de novo variant was classified based on American College of Medical Genetics and Genomics (ACMG)/Association for Molecular Pathology (AMP) criteria (40).

### Digenic combinations in haploinsufficient genes.

Haploinsufficient genes were identified based on a pLI score greater than 0.9, obtained from the Genome Aggregation Database (gnomAD), comprising 141,456 human genomes (18). Genes located in sex chromosomes were excluded, resulting in a final set of 2,831 autosomal genes (chromosomes 1–22) for further analysis. To ensure reliable results, we utilized an exploratory and validation cohort consisting of 52 trios and 69 proband-only samples. Digenic combinations (gene A × gene B) with rare variants (minor allele frequency [MAF] < 0.5%) in haploinsufficient genes, specifically LGD or D-mis variants, were prioritized. Using the 52 trios, we searched for all possible combinations, considering 1 allele from the father and 1 allele from the mother. Digenic pairs that also contained LGD or D-mis rare variants in the parents or population controls were excluded to minimize false-positive detections. Consequently, we extracted case-specific digenic combinations harboring LGD or D-mis rare variants. To assess the pathogenicity of these digenic combinations, we employed the Variant Combination Pathogenicity Predictor (VarCoPP) provided by the Oligogenic Resource for Variant Analysis (ORVAL) (16). VarCoPP utilizes individual variant information to classify digenic combinations into 4 categories: “Neutral,” “Candidate disease-causing,” “99%-zone disease-causing,” and “99.9%-zone disease-causing,” based on the VarCoPP score. We focused exclusively on the predicted disease-causing combinations falling within the 99.9%-zone. Additionally, we considered genes with reported brain or skeletal expression in the Genotype-Tissue Expression (GTEx) portal (https://gtexportal.org/) (41). To validate the selected digenic combinations, we additionally analyzed the 69 proband-only samples.

### PCR and Sanger sequencing.

To validate each variant that resulted from exome sequencing, a DNA sample from each patient was extracted using the Quick-DNA Miniprep Plus Kit (D4068, Zymo Research). PCR primers were designed for each location, and PCR was conducted. Sanger sequencing was performed on each PCR result. The primer information for each gene is provided in Supplemental Table 7.

### Dual-luciferase promoter activity assay.

To investigate the functional activities of *TRPS1* and *IL6ST* gene variants, we conducted in vitro experiments using a Dual-Luciferase Reporter Assay System (E1910, Promega). Plasmids encoding C-terminal DYK-tagged human TRPS1 (RC215856, OriGene) and IL6ST (RC215123, OriGene) were commercially purchased for this study. We generated *TRPS1* (Q181R and R814L) and *IL6ST* (N360S and S580F) mutants from WT vectors using site-directed mutagenesis techniques. These mutants were used to assess the functional activities of the variants. To eliminate the potential confounding effect of endogenous *TRPS1* and *IL6ST* expression, we selected HepG2 and HEK293 cells, respectively, for the assay (Supplemental Figure 7). HepG2 cells were plated and allowed to attach for 24 hours prior to transfection, which was performed using TransIT-LT1 Transfection Reagent (MIR2300, Mirus Bio). We utilized the p6OSE2-luciferase vector (200 ng/24 well) to measure the effect of TRPS1 on osteogenic-specific function. To assess luciferase activity, we cotransfected the RUNX2 vector (250 ng/24 well) with the*TRPS1* WT, Q181R, or R814L variant (30 ng/24 well). For *IL6ST* variants, HEK293 cells were plated and allowed to attach for 24 hours. To eliminate endogenous *IL6ST* expression, we employed IL6ST siRNA to silence its expression. The siRNA specifically targeted the 3′-UTR of *IL6ST* mRNA and was transfected using TransIT-LT1 Transfection Reagent. After 6 hours, we transfected *IL6ST* variants (WT, N360S, and S580F) at a concentration of 200 ng/24 well. We used pGL4.47[*luc2P*/SIE/Hygro] (E4041, Promega), IL11RA (RC200654, OriGene), and pGL4.70[*hRluc*] (E6881, Promega) vectors to measure the activities of the IL6ST variants. Following transfection, the cells were maintained in a serum-free medium and stimulated with recombinant human IL-11 (1 ng/mL) (P20809, PeproTech) for 6 hours. Luciferase assay results were normalized by assessing *Renilla* luciferase activity and presented as Firefly/*Renilla* luciferase activity.

### Double knockdown of Trps1 and Il6st in murine cells.

To investigate the combined effects of *TRPS1* and *IL6ST* on bone ossification, we performed a double knockdown experiment using MC3T3-E1 mouse osteoblasts (ATCC CRL-2593). The cells were cultured in 12-well plates using α-MEM without l-ascorbic acid (LM008-53, Welgene), supplemented with 10% FBS (26140-079, Gibco), and penicillin/streptomycin (15140-122, Gibco) during the cell proliferation phase. During the differentiation phase, the culture medium was supplemented with 50 μg/mL l-ascorbic acid (A4544, MilliporeSigma) and 10 mM β-glycerophosphate (G9422, MilliporeSigma), and replaced every 3 days. After the MC3T3-E1 cells were plated in a 12-well plate for 16–24 hours, siRNAs targeting *Trps1* and *Il6st* (Supplemental Table 7) were transfected into the cells using Lipofectamine RNAiMAX (13778-100, Invitrogen) as per the manufacturer’s protocol. The cells were allowed to grow and stabilize for 48 hours after transfection to ensure proper cell growth and environment. Osteogenic induction medium was then added and replaced every 3 days. After day 5–7 of osteogenic induction, the cells were extracted from the wells using Tri-RNA Reagent (FATRR001, FAVORGEN), and the resulting cell lysates were used for the ALP assay and qRT-PCR. To investigate the roles of TRPS1 and IL6ST in osteoclasts, we utilized the RAW 264.7 cell line (ATCC TIB-71). The cells were seeded in 24-well plates using α-MEM (LM008-01, Welgene), supplemented with 10% FBS and penicillin/streptomycin. After seeding for 16–24 hours, siRNAs targeting *Trps1* and *Il6st* (Supplemental Table 7) were transfected into the cells using TransIT-LT1 Transfection Reagent. After 24 hours, to induce osteoclast differentiation, 100 ng/mL RANKL (315-11, PeproTech) was added to the cells every day. For qRT-PCR, the cells were extracted day 3 after the start of differentiation. After day 5 of differentiation, TRAP staining was performed.

### ALP assay, staining, and quantitative RT-PCR.

To measure ossification of osteoblasts, we used the ALP assay kit (ab83369, Abcam) following the manufacturer’s protocol. In brief, cell lysates were collected from the ALP assay buffer in 12-well plates, and *p*-nitrophenyl phosphate (pNPP) solution was added to 96-well plates. After addition of the pNPP solution to each well, the plates were incubated in the dark for 1 hour. ALP activity was measured based on colorimetric changes using an ELISA reader. For ALP staining, we used the ALP staining kit (294-67001, Wako). After day 5~7 of osteoblast differentiation, the culture medium in each well was removed and washed using D-PBS (LB001-02, Welgene). Each well was then treated with formaldehyde for 10 minutes in an icebox and fixed by alcohol/acetone, and the ALP staining solution was added. After 15 minutes, ALP staining was visually confirmed. To extract cell lysates, we used the Tri-RNA Reagent. Total RNA was isolated from the cell lysates using the RNA extraction kit (K-3140, Bioneer) in accordance with the manufacturer’s instructions. RNA-to-cDNA conversion was performed using the EcoDry Premix (639549, Takara) following the recommended protocol. To assess the knockdown efficiency of the target genes (*Trps1* and *Il6st*) after 48 hours of transfection, qRT-PCR was performed. Additionally, after day 7 of osteoblast differentiation, bone formation markers (*Ibsp*, *Bglap1*, *Col1a1*, *Runx2*, *Alpl*) were analyzed to determine the extent of differentiation**)**. Primer information for each gene is provided in Supplemental Table 7.

### TRAP staining, multinucleated cell count, and qRT-PCR.

TRAP staining was performed using the TRAP staining kit (294-67001, Wako). After day 5 of osteoclast differentiation, the culture medium in each well was removed and washed with D-PBS (LB001-02, Welgene), following the same preparation process as described above for ALP staining. The TRAP staining solution was added, and staining was allowed to proceed for 15 minutes. To quantify the TRAP staining, multinucleated cells — defined as those containing 5 or more nuclei — were counted. The number of counts was converted to percentages based on the scrambled group. To assess the knockdown efficiency of *Trps1* and *Il6st* after 24 hours of transfection, qRT-PCR was performed. Furthermore, after day 3 of osteoclast differentiation, osteoclast markers (*Ctsk*, *Dcstamp*, *Trap*, and *Nfatc1*) were analyzed to assess differentiation using qRT-PCR. Primer information for each gene is provided in Supplemental Table 7.

### Western blot analysis.

To perform Western blot analysis, we cultured MC3T3E1 cells in 12-well plates and washed them twice with D-PBS. The cells were then scraped in Pierce RIPA buffer (89900, Thermo Fisher Scientific) and incubated for 6 hours in osteogenic induction medium. Following incubation, the lysates were sonicated for 20 seconds and centrifuged at 13,200*g* for 15 minutes at 4°C. The protein concentration of the resulting supernatants was determined using a BCA Protein Assay Kit (T9300A, Takara). Equal amounts of protein were loaded onto a 10% SDS-PAGE gel and subsequently transferred to a nitrocellulose membrane (10600004, Amersham). To minimize nonspecific binding, the membrane was blocked with either 5% skim milk or 5% BSA in TBS buffer for 1 hour at room temperature. Subsequently, the membrane was incubated overnight with primary monoclonal antibodies. Following the washing process, the membrane was incubated with a secondary monoclonal antibody, and the protein bands were visualized using a film. The intensity of the protein bands was quantified using the Densitometry program (Multi Gauge V3.0, Fujifilm).

### Differential expression of Il6st and Trps1 in calvarial sutures and long bones.

C57BL/6 mice (1 and 8 weeks old) were purchased from Orient Bio and euthanized by CO_2_ asphyxiation. Immediately following euthanasia, mice were dissected to collect calvarial sutures, femurs, and cartilage. Whole sutures, femurs, and cartilage were harvested from mice at 1 and 8 weeks of age. The entire sutures were dissected in detail into lambdoid, metopic, coronal, and sagittal regions. Tissues were sonicated in Tri-RNA Reagent for 1 minute at 30% amplitude for RNA extraction. RNA was isolated using Direct-zol RNA Miniprep Plus Kits (R2073, Zymo Research) according to the manufacturer’s instructions. RNA was converted to cDNA using EcoDry Premix (639549, Takara) and analyzed by quantitative RT-PCR to quantify *Ilst1* and *Trps1* gene expression.

### Primary cultures of mouse osteoblasts and siRNA transfection.

Pregnant C57BL/6 mice at 18 days of gestation were used to obtain embryos for analysis. Mice were euthanized by CO_2_ asphyxiation, following American Veterinary Medical Association (AVMA) guidelines. Following euthanasia, the mouse calvaria was carefully dissected to extract the embryos for immediate experimental use. Six to 8 calvariae were harvested from the embryos and washed with D-PBS (LB001-02, Welgene). Each calvaria was then incubated in 10 mL of a solution containing 10 mg Collagenase A (10103578001, Merck) dissolved in 100 mL α-MEM without l-ascorbic acid (LM008-53, Welgene) at 37°C with shaking for 20 minutes. The solution was discarded, and the calvariae were gently rinsed with another 10 mL fresh enzyme solution without disturbing the calvariae. This process was repeated twice, and the third enzyme solution was filtered through a cell strainer (93070, SPL). The filtrate was centrifuged at 1,600 x g for 5 minutes to pellet the cells. This procedure was repeated twice more, accumulating 3 separate cell pellets. The pellets were then pooled and cultured together in α-MEM) supplemented with 10% FBS (26140-079, Gibco) and penicillin/streptomycin (15140-122, Gibco) without l-ascorbic acid. Cells were maintained in 150 mm tissue culture dishes (93150, TPP) and not passaged beyond passage 5. During the differentiation period, the medium was supplemented with 50 μg/mL l-ascorbic acid (A4544, MilliporeSigma) and 10 mM β-glycerophosphate (G9422, MilliporeSigma), and refreshed every 3 days. Primary osteoblastic cells were seeded in 24-well plates. Sixteen to 24 hours after seeding, cells were transfected with siRNAs targeting *Trps1* and *Il6st* (Supplemental Table 7) using Lipofectamine RNAiMAX (13778-100, Invitrogen) according to the manufacturer’s protocol. After transfection, cells were allowed to stabilize for 24 hours before addition of osteogenic induction medium, which was replaced every 3 days. Osteogenic induction was carried out for 5–7 days, after which cells were harvested using Tri-RNA Reagent (FATRR001, FAVORGEN) for subsequent qRT-PCR analysis.

### Structural modeling.

The AlphaFold Protein Structure Database was used to retrieve the predicted protein structure of *TRPS1* (UniProt: Q9UHF7) and *IL6ST* (UniProt: P40189) (42). The PyMOL program (v.2.5.5; PyMOL Molecular Graphics System, Schrödinger) was used to visualize the figures.

### Statistics.

Statistical analyses were performed using the software R (version 4.1.2). A *P* value less than 0.05 was considered significant. Data were analyzed using the Mann-Whitney *U* test or 1-way ANOVA followed by Tukey’s multiple-comparison test, as appropriate. Protein-protein interaction network and pathway enrichment analyses were conducted using the STRING database (https://string-db.org/) (43) and ShinyGO 0.77 (44).

### Study approval.

Informed consent was obtained from all participants or their parents. This study was conducted in accordance with the principles outlined in the Declaration of Helsinki and was approved by the local ethics committee (Yonsei University Health System 4-2015-0676). All procedures involving mouse experiments were conducted in accordance with the guidelines of the Institutional Animal Care and Use Committee (IACUC) and were approved by the IACUC of Yonsei University Health System (Approval No. 2020-0153 and 2023-0167).

### Data availability.

Most of the data are provided in the present manuscript. Due to restrictions relating to informed consent, exome data may not be shared in secure access–controlled repositories. Such data are available from the corresponding author upon reasonable request. Korea1K whole-genome sequencing is available at http://1000genomes.kr/ Values for all data points in graphs are reported in the Supporting Data Values file.

## Author contributions

JWY, JGY, and MGL conceptualized and designed the study. JWY, JGY, and CH analyzed data and prepared figures. JWY, CH, SHN, and YMY performed experiments and analyzed data. DMS, YOK, and KWS collected the clinical samples. All authors provided intellectual input and reviewed and approved the final manuscript. JWY and JGY contributed equally as co–first authors. Between the 2, authorship order was decided as follows: JWY performed the majority of the experiments and data acquisition, while JGY conducted bioinformatics analysis and prepared the manuscript.

## Supplementary Material

Supplemental data

Unedited blot and gel images

Supporting data values

## Figures and Tables

**Table 1:**
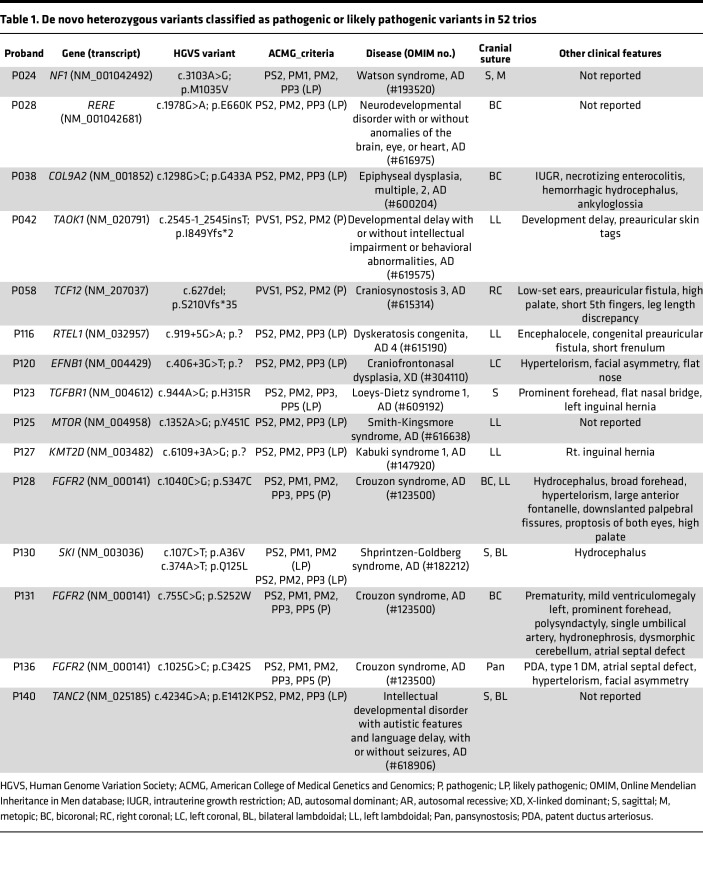
De novo heterozygous variants classified as pathogenic or likely pathogenic variants in 52 trios

**Table 2:**
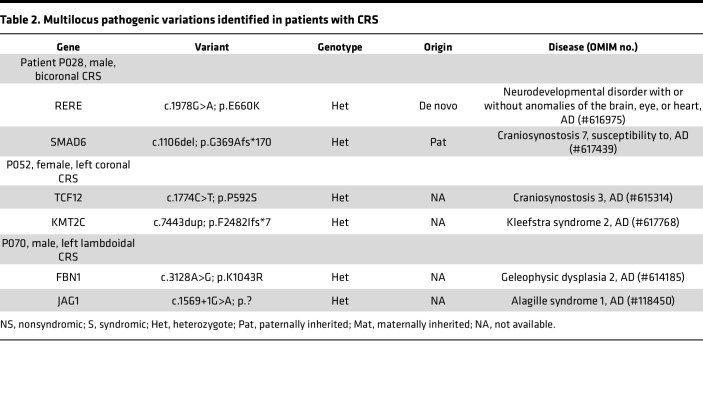
Multilocus pathogenic variations identified in patients with CRS

## Version 1 02/24/2025

Electronic publication
