# Supplementary material for: Digenic impairments of haploinsufficient genes in patients with craniosynostosis
Source: JCI Insight. 2025 Feb 24;10(4):e176985. doi: 10.1172/jci.insight.176985 (PMC11949007; doi:10.1172/jci.insight.176985)
Supplement: Supplemental data [file jciinsight-10-176985-s042.pdf]

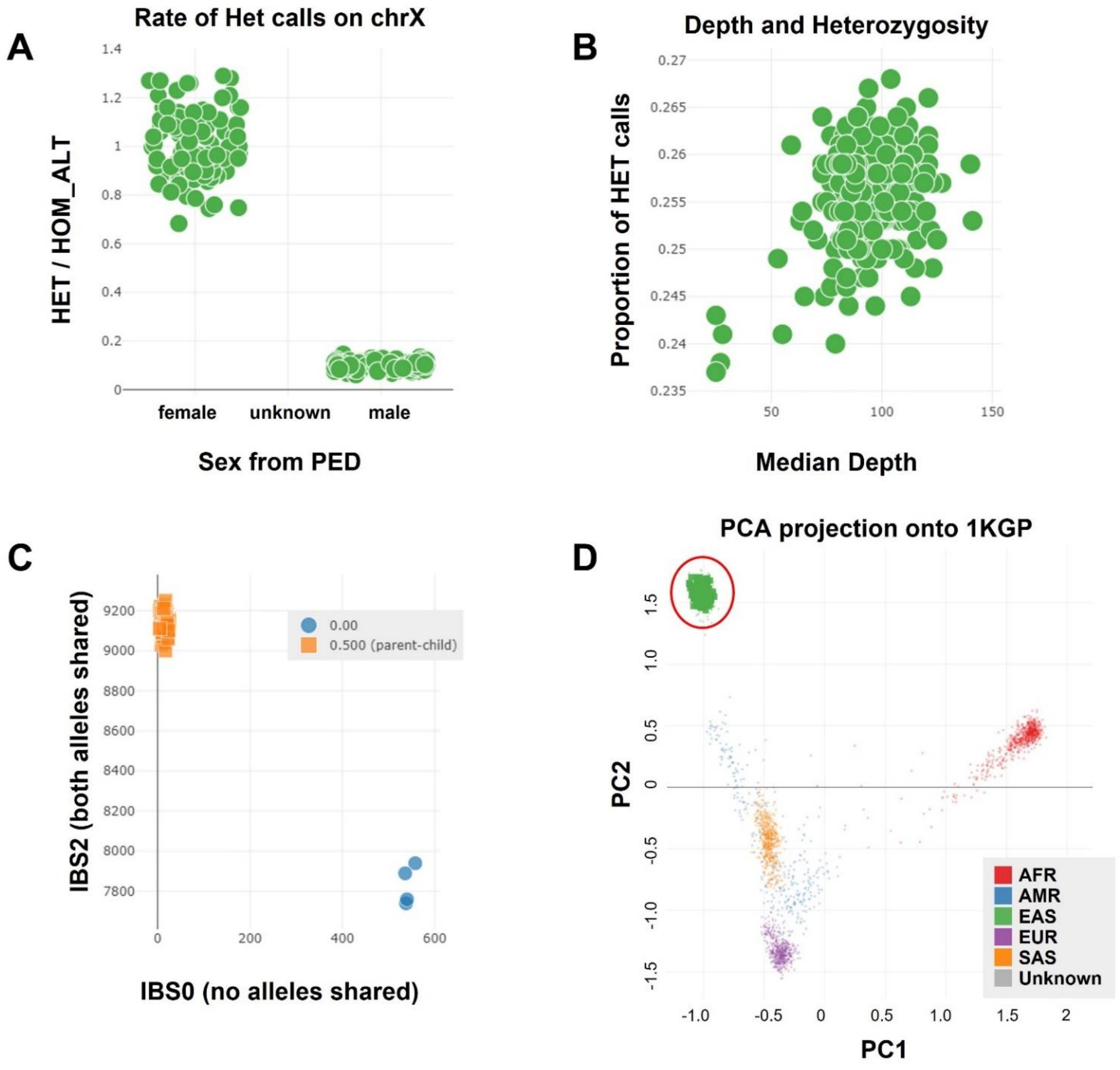

Supplemental Figure 1. Quality control of exome sequencing data using *Peddy*.

(A) Comparison of reported sex and the rate of heterozygous calls on the X chromosome demonstrates identical matching, indicating no violation of sex. **(B)** Distribution of median depth for the heterozygous calls shows high coverage, indicating that sequencing depth is sufficient for accurate variant calling. **(C)** Relatedness checks using the identity-by-state (IBS) plot in the probands and parents show expected results, indicating accurate sample identification and relatedness determination. **(D)** Ancestry prediction on the principal component analysis (PCA) plot indicates that all participants are of East Asian ancestry. No violations of these quality control measures were observed, indicating that the 225 exome sequencing data are suitable for downstream analyses.

A

Variant type	observed	expected	enrichment	*P* value
Syn	14	14.6	0.96	0.596
Mis	54	32.8	1.65	0.000412
LGD	7	4.5	1.54	0.175
LGD + Mis	61	37.3	1.64	0.000227

B

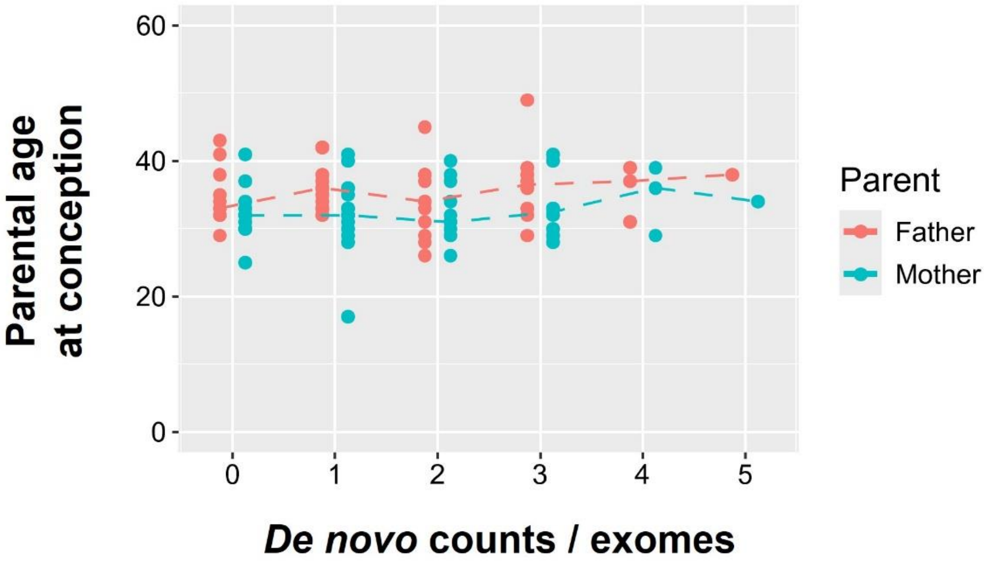

Supplemental Figure 2. ***De novo*** **variant analysis in 52 trios.**

De novo variant analysis in 52 trios reveals 80 *de novo* protein-coding variants, including 76 single nucleotide variants (SNVs) and four insertions and deletions. **(A)** Burden analysis using denovolyzeR shows that our patients have high burdens of protein-altering *de novo* variants, with 1.65-fold enrichment in missense variants ($P = 4.1 \times 10^{-4}$). **(B)** The *de novo* variant counts per exome did not exhibit significant differences according to the parental ages, although the limited number of cases should be acknowledged.

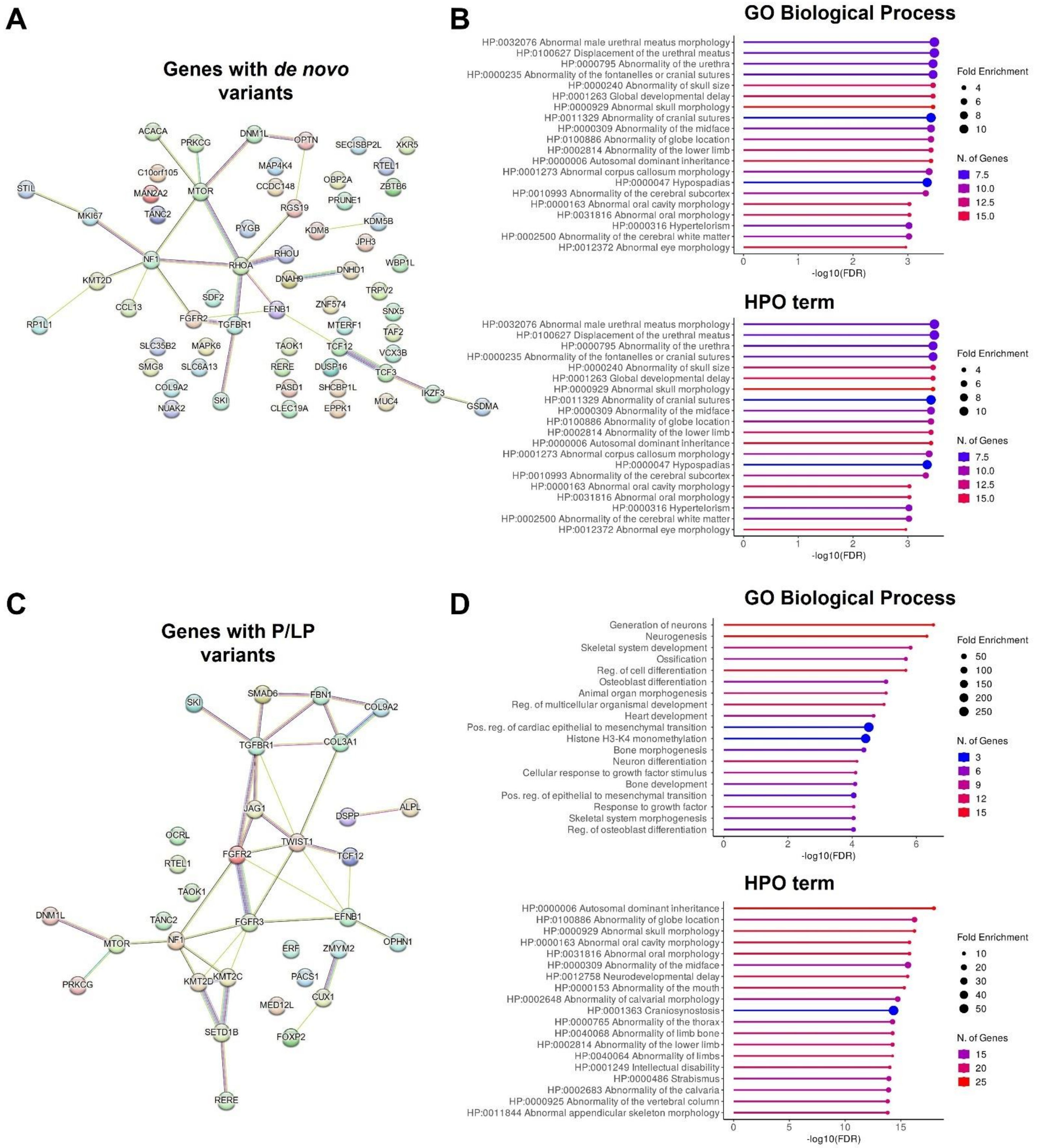

Supplemental Figure 3. Gene network and pathway enrichment analyses of genes associated with craniosynostosis.

Gene network and pathway enrichment analyses were conducted on the genes that have functionally altered (likely gene disrupting or missense) *de novo* variants **(A, B)** or pathogenic (P) or likely pathogenic (LP) variants **(C, D)**. The protein-protein interaction networks of **(A)** the 61 genes with *de novo* variants and **(C)** the 33 genes with P/LP variants show that known craniosynostosis (CRS) genes are frequently located on the cores among many interactions at the molecular level.

Pathway enrichment analysis shows that there are significant enrichments of **(B)** the 61 genes with *de novo* variants and **(D)** the 33 genes with P/LP variants in the Gene Ontology (GO) biological processes related to Neurogenesis (GO:0022008) and Ossification (GO:0001503), and human phenotype ontology (HPO) terms related to Abnormality of the fontanelles or cranial sutures (HPO:0000235) and Abnormal skull morphology (HPO:0000929).

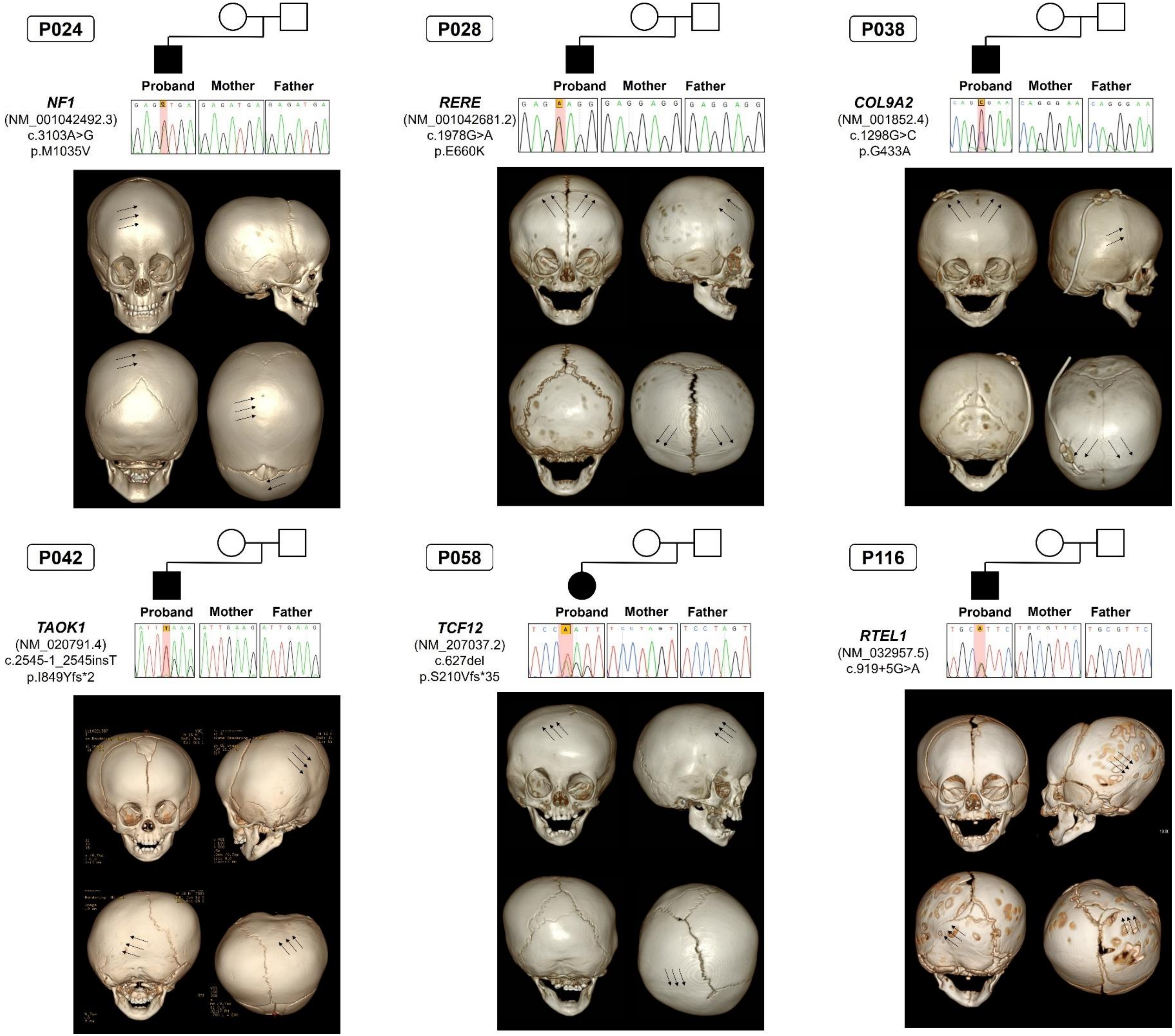
P024
Proband
Mother
Father
NF1
(NM_001042492.3)
c.3103A>G
p.M1035V
P028
Proband
Mother
Father
RERE
(NM_001042681.2)
c.1978G>A
p.E660K
P038
Proband
Mother
Father
COL9A2
(NM_001852.4)
c.1298G>C
p.G433A
P042
Proband
Mother
Father
TAOK1
(NM_020791.4)
c.2545-1_2545insT
p.I849Yfs*2
P058
Proband
Mother
Father
TCF12
(NM_207037.2)
c.627del
p.S210Vfs*35
P116
Proband
Mother
Father
RTEL1
(NM_032957.5)
c.919+5G>A

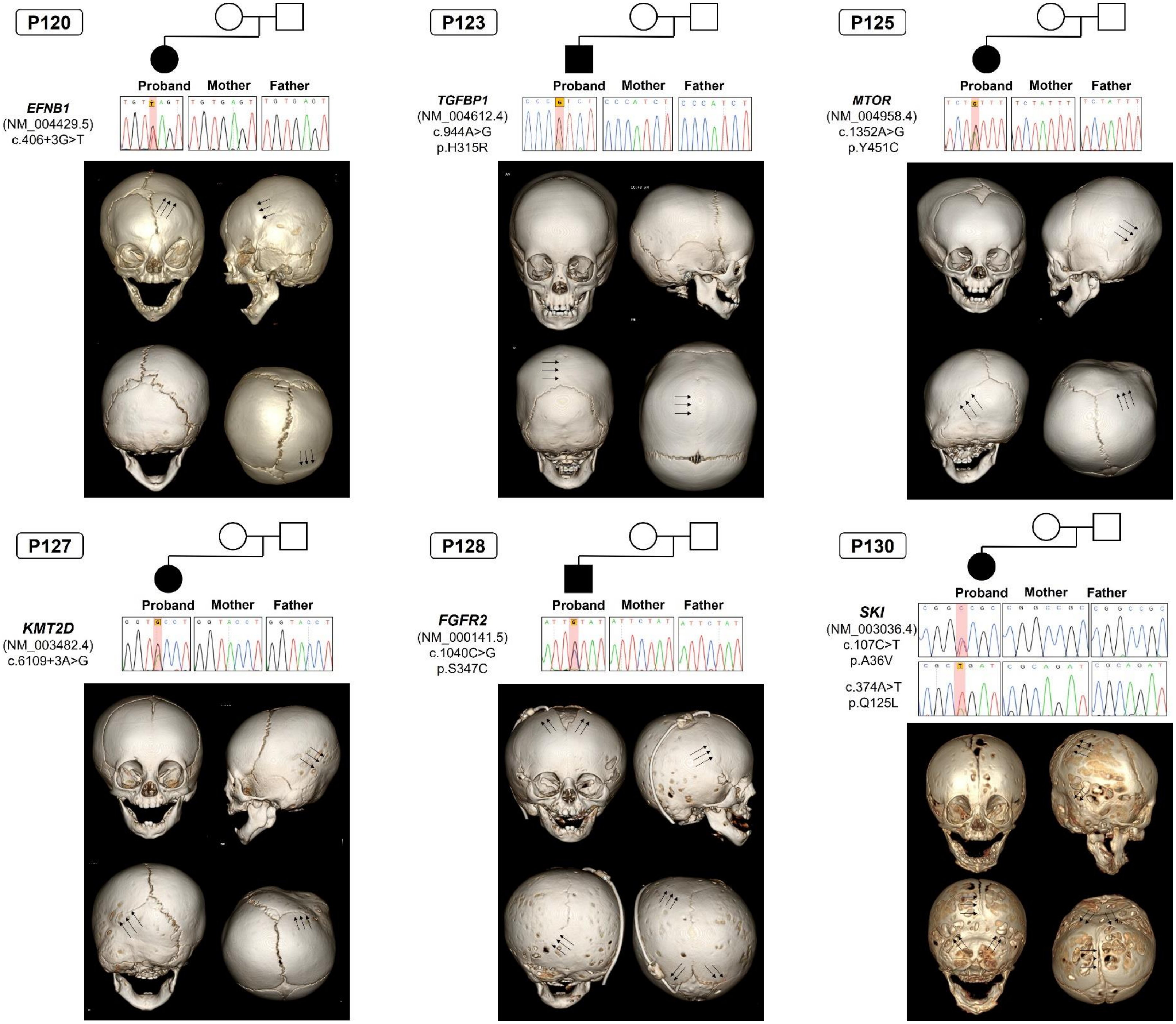

P120
Proband
Mother
Father
EFNB1
(NM_004429.5)
c.406+3G>T
P123
Proband
Mother
Father
TGFBP1
(NM_004612.4)
c.944A>G
p.H315R
P125
Proband
Mother
Father
MTOR
(NM_004958.4)
c.1352A>G
p.Y451C
P127
Proband
Mother
Father
KMT2D
(NM_003482.4)
c.6109+3A>G
P128
Proband
Mother
Father
FGFR2
(NM_000141.5)
c.1040C>G
p.S347C
P130
Proband
Mother
Father
SKI
(NM_003036.4)
c.107C>T
p.A36V
c.374A>T
p.Q125L

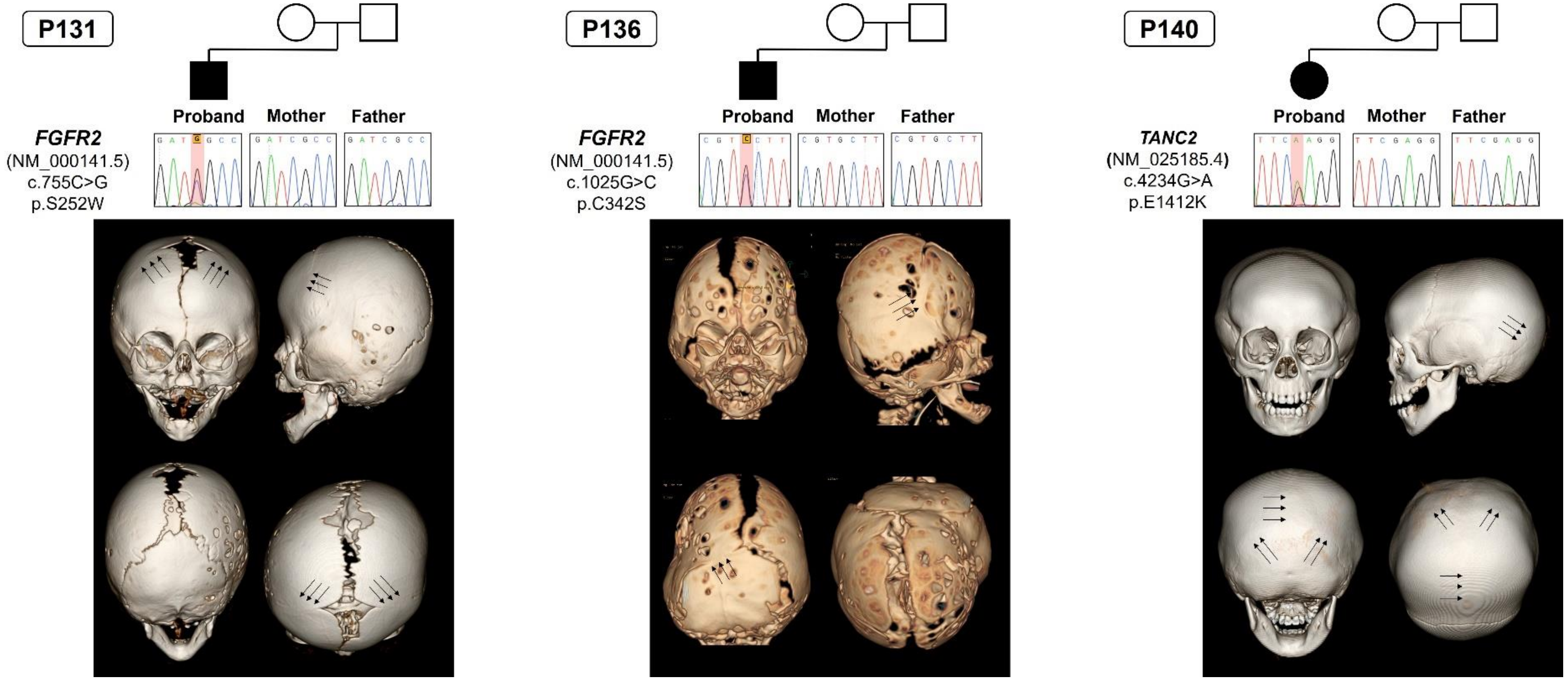

Supplemental Figure 4. Validation of *de novo* variants and three-dimensional skull CT scan reconstructions.

Three-dimensional reconstructions of skull computed tomography (CT) scans are presented for each patient, as detailed in **Table 1**. Additionally, *de novo* variants identified from exome sequencing were validated through Sanger sequencing analysis. Genomic DNA was extracted from the patient and their respective parents' blood samples and subjected to quality control processes. Data for P011 was published elsewhere.

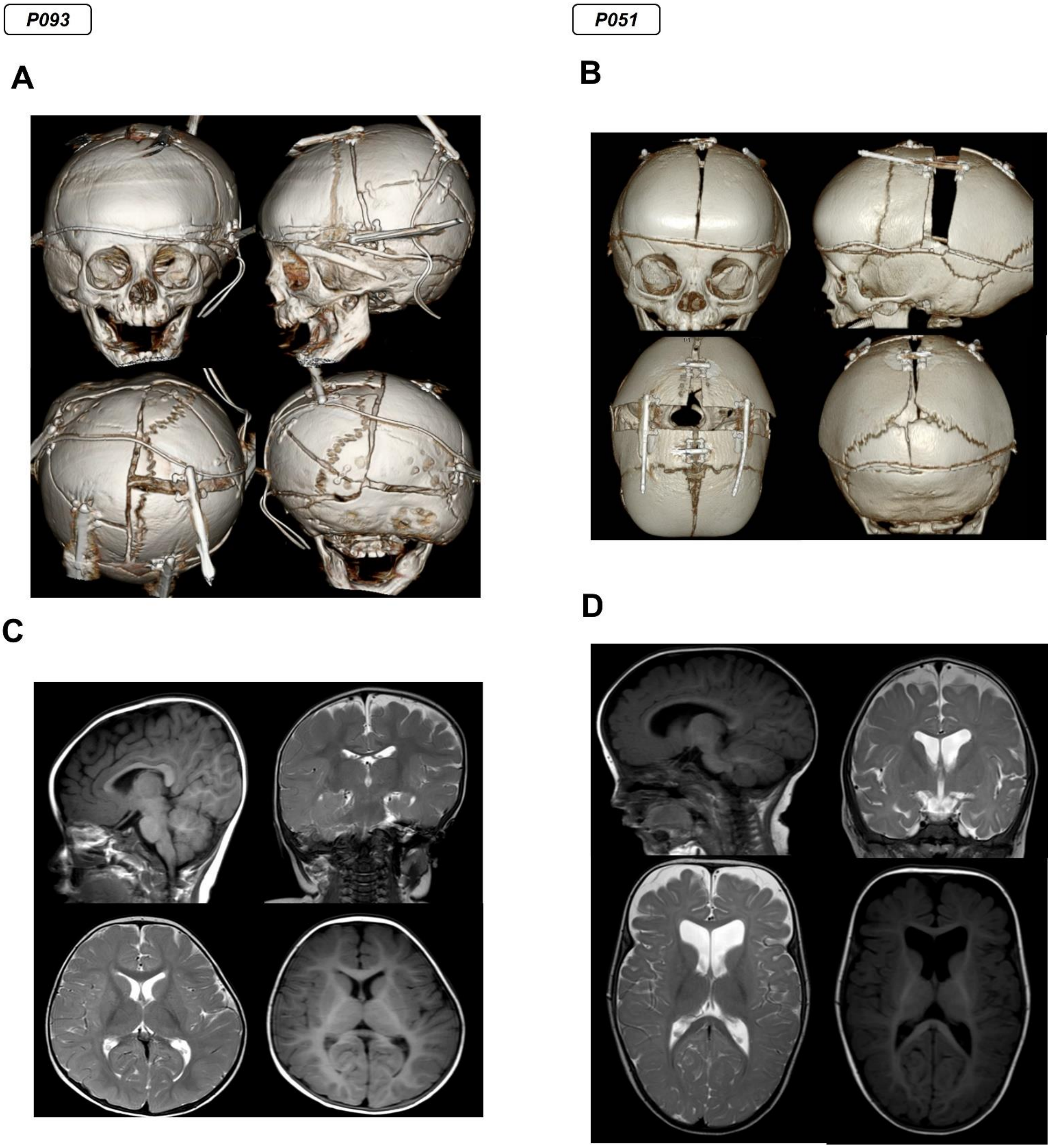

Supplemental Figure 5. Clinical information of patients with *TRPS1* and *IL6ST* variants.

The figure depicts clinical data of two craniosynostosis patients (P051, P093) with no family history of the condition. Skull deformity was identified after 4-6 months of birth, leading to diagnosis through Skull X-ray and CT imaging. Distraction osteogenesis was performed within the first year of birth (P051: 6 months, P093: 11 months) without any complications. Post-surgery CT **(A, B)** and MRI images **(C, D)** showed successful correction and normal brain structure, and continuous follow-up indicated average development in both patients.

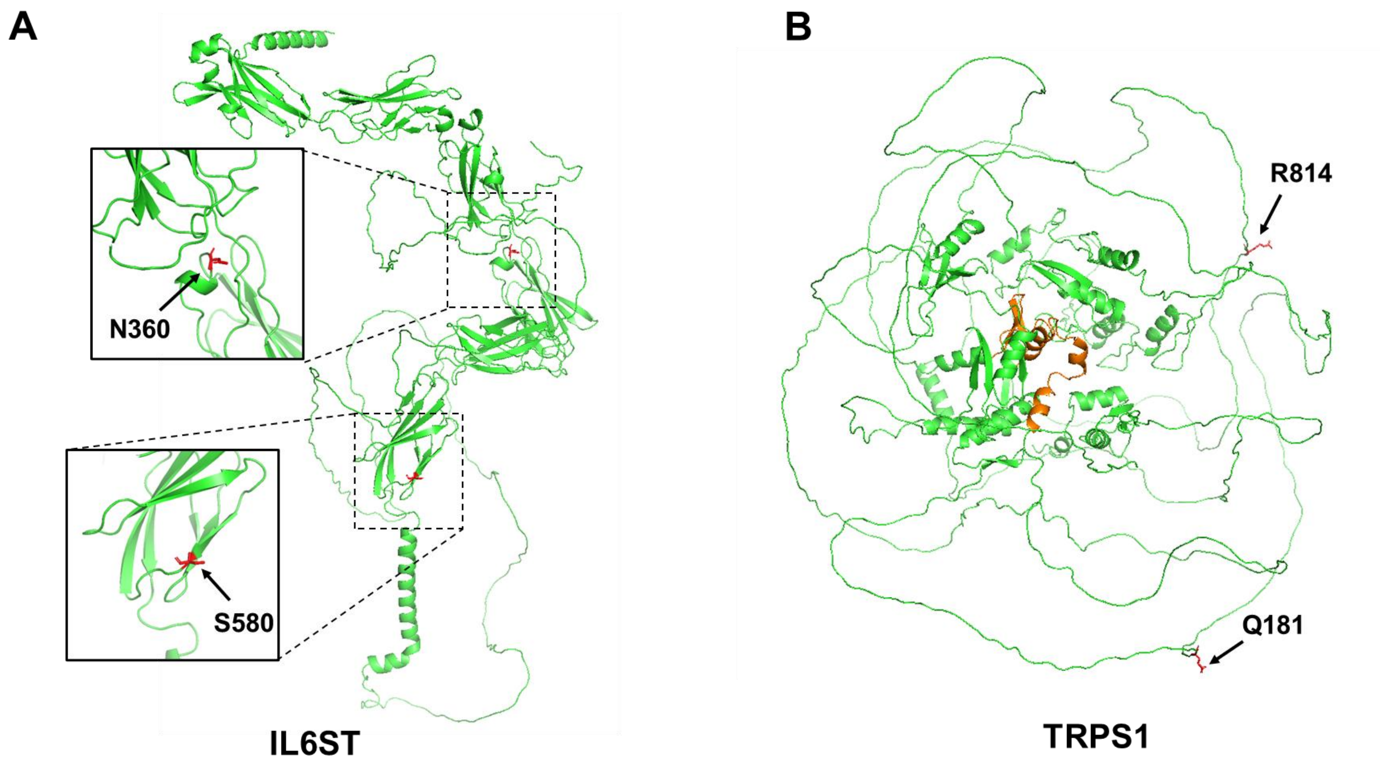

Supplemental Figure 6. Localization of the identified variants in the predicted protein 3D structures of TRPS1 and IL6ST using AlphaFold.

(A) The transmembrane protein GP130, encoded by IL6ST, is depicted with two variants (N360S and S580F) located in extracellular domains. These variants exhibited reduced activities compared to the wild-type. **(B)** TRPS1, a nuclear transcription factor, was partially predicted by AlphaFold. The core structures, including the GATA-type Zn finger domain highlighted in orange, were well-predicted. The two variants (Q181R and R814L) were found outside the GATA-type Zn finger domain, which is associated with trichorhinophalangeal syndrome. These variants showed lower transcriptional repression activities than the wild-type.

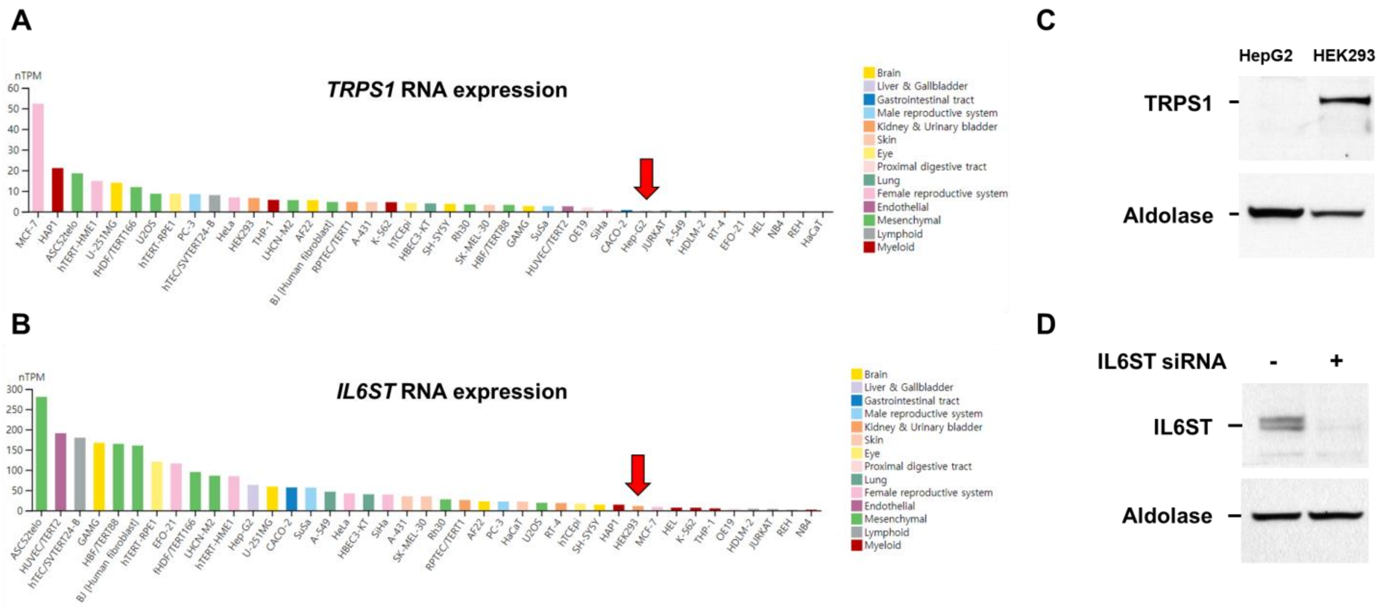

Supplemental Figure 7. Expression levels of *TRPS1* and *IL6ST* in different tissues and cell lines for assessment of variant activities.

(A) Expression levels of *IL6ST* across various tissues based on GTEx data. Due to the widespread expression of *IL6ST*, HEK293 cells were chosen, and endogenous expression was downregulated using siRNA. **(B)** Expression levels of *TRPS1* across various tissues based on GTEx data. Liver tissues (red arrow) exhibited the lowest *TRPS1* expression levels, prompting the selection of HepG2 cells for the evaluation of TRPS1 mutant activity. **(C)** Western blot analysis of TRPS1 protein levels in HepG2 and HEK293 cell lines. The results confirm the absence of endogenous *TRPS1* expression in HepG2 cells. **(D)** Knockdown of endogenous *IL6ST* expression in HEK293 cells using siRNA against the 3'-untranslated region of *IL6ST*. Western blot results demonstrate efficient suppression of *IL6ST* expression via siRNA treatment.

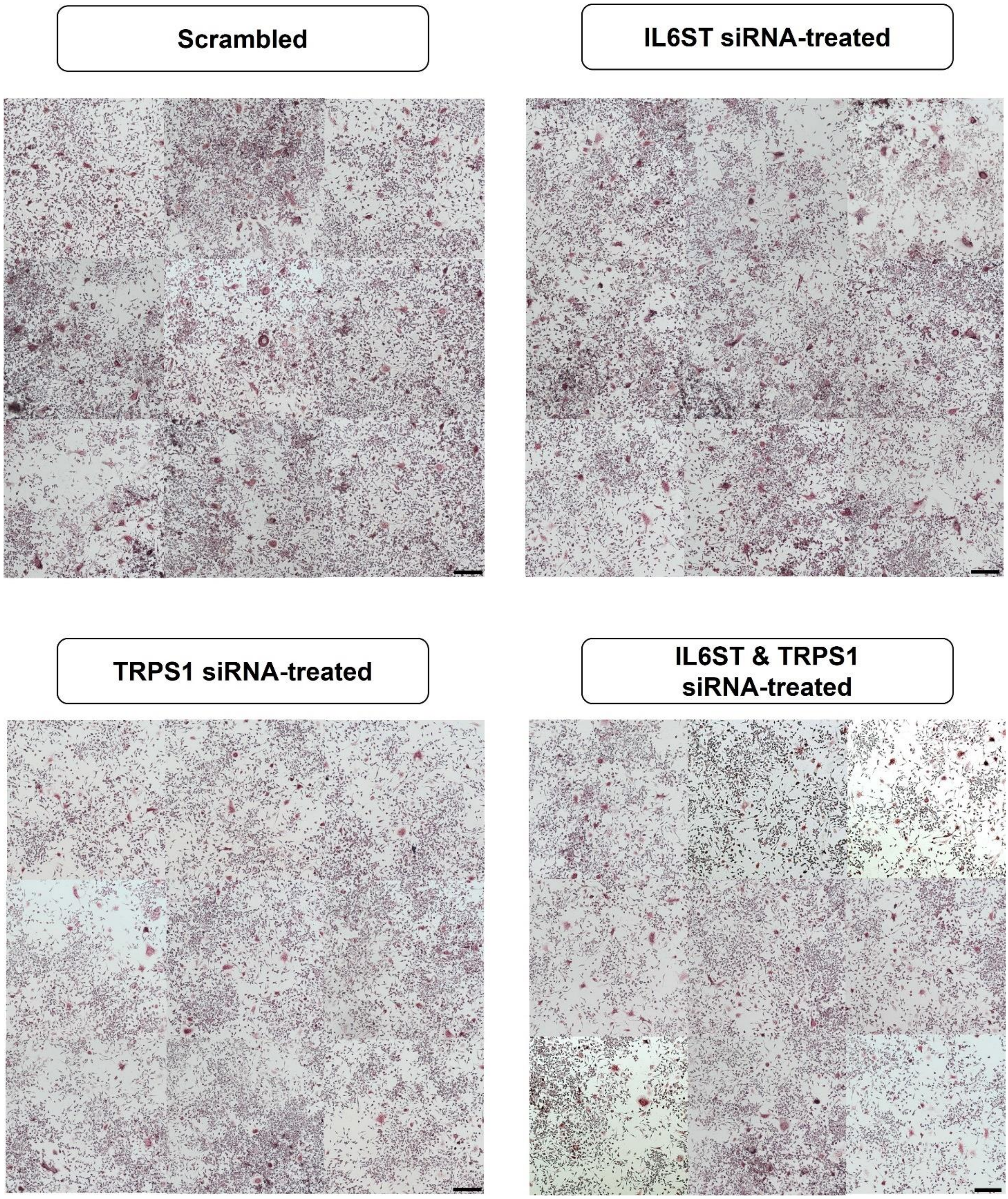

Supplemental Figure 8. Representative images of tartrate-resistant acid phosphatase (TRAP) staining to assess osteoclast differentiation.

TRAP staining was performed on day 5 after initiation of differentiation to evaluate osteoclast formation. Images were captured at 9 different locations per well and subsequently combined. The quantification of multi-nucleated cells was conducted on biologically independent samples (*n* = 3). The scale bar represents 100 µm.

Supplemental Table 1. Characteristics of 121 Korean patients with craniosynostosis.

**Characteristics**		***n***	**%**
Age	month, median [range]	15	[2-120]
Sex			
	Male	74	61.2%
	Female	47	38.8%
Exome sequencing			
	Trio	52	43.0%
	Proband only	69	57.0%
Molecular assessment			
	Targeted sequencing for 34 CRS genes	93	76.9%
	Unsolved cases	79	65.3%
	Prior diagnosis[a]	14	11.6%
	No prior molecular work-up	28	23.1%
Clinical features			
	Syndromic	44	36.4%
	Non-syndromic	77	63.6%
Cranial suture involvement			
	Metopic (M)	9	7.4%
	Sagittal (S)	43	35.5%
	Coronal (C)	34	28.1%
	Lambdoid (L)	16	13.2%
	Multiple	19	15.7%

[a]Cases with chromosomal abnormalities, including 1p32-p31 deletion (*n* = 1), 10q26 deletion (*n* = 1), 16p11.2 deletion (*n* = 2), 17p13.3 deletion (*n* = 1), and incomplete penetrance genes, including *TCF12* (*n* = 4), *ERF* (*n* = 3), *ALPL* (*n* = 1), *FBN1* (*n* = 1), were enrolled.

Supplemental Table 2. Complete list of *de novo* variants detected in 52 trios.

Sample	Transcript	Gene	HGVS coding	HGVS protein	Zygosity
P002	NM_030640	*DUSP16*	c.1087G>A	p.V363M	Het
P005	NM_014426	*SNX5*	c.874A>T	p.T292S	Het
P008	NM_003223	*TFAP4*	c.702G>A	p.T234=	Het
P009	NM_183008	*UBXN11*	c.690C>T	p.L230L	Het
P011	NM_024773	*KDM8*	c.590A>G	p.E197G	Het
P011	NM_004834	*MAP4K4*	c.569G>T	p.G190V	Het
P023	NM_030933	*SHCBP1L*	c.898G>A	p.A300T	Het
P023	NM_031308	*EPPK1*	c.367C>A	p.P123T	Het
P024	NM_001083913	*WBP1L*	c.104G>T	p.C35F	Het
P024	NM_001042492	*NF1*	c.3103A>G	p.M1035V	Het
P028	NM_001042681	*RERE*	c.1978G>A	p.E660K	Het
P028	NM_003200	*TCF3*	c.1651C>T	p.R551W	Het
P035	NM_001048166	*STIL*	c.1473G>T	p.Q491H	Het
P038	NM_001852	*COL9A2*	c.1298G>C	p.G433A	Het
P038	NM_001372	*DNAH9*	c.4615G>A	p.D1539N	Het
P038	NM_138803	*CCDC148*	c.1265G>T	p.W422L	Het
P041	NM_020245	*TULP4*	c.1020C>T	p.L340=	Het
P041	NM_178857	*RP1L1*	c.1035G>T	p.R345S	Het
P042	NM_002417	*MKI67*	c.4793G>A	p.R1598Q	Het
P042	NM_020791	*TAOK1*	c.2545-1_2545insT	p.I849Yfs*2	Het
P055	NM_006923	*SDF2*	c.373G>T	p.G125C	Het
P055	NM_002739	*PRKCG*	c.641C>T	p.T214M	Het
P058	NM_207037	*TCF12*	c.627del	p.S210Vfs*35	Het
P059	NM_030952	*NUAK2*	c.742A>G	p.M248V	Het
P059	NM_022752	*ZNF574*	c.1949G>A	p.R650Q	Het
P062	NM_198925	*SEMA4B*	c.2353C>A	p.R785=	Het
P074	NM_002862	*PYGB*	c.2443G>T	p.D815Y	Het
P090	NM_002748	*MAPK6*	c.1603A>G	p.I535V	Het
P091	NM_012062	*DNM1L*	c.1073C>T	p.S358L	Het
P091	NM_012481	*IKZF3*	c.1160C>T	p.T387M	Het
P091	NM_018406	*MUC4*	c.12263C>T	p.S4088L	Het
P093	NM_006825	*CKAP4*	c.648G>A	p.S216=	Het
P093	NM_178171	*GSDMA*	c.1081G>A	p.V361M	Het
P093	NM_006980	*MTERF1*	c.1138C>T	p.L380F	Het
P093	NM_006626	*ZBTB6*	c.1075T>A	p.C359S	Het
P097	NM_152598	*MARCHF10*	c.2121C>T	p.A707=	Het
P112	NM_014671	*UBE3C*	c.252C>T	p.G84=	Het
P112	NM_003184	*TAF2*	c.1352G>A	p.C451Y	Het
P112	NM_173493	*PASD1*	c.1190A>G	p.N397S	Het
P116	NM_032957	*RTEL1*	c.919+5G>A	p.?	Het
P117	NM_001039467	*RGS19*	c.83C>T	p.T28I	Het
P119	NM_001193489	*SECISBP2L*	c.2341C>T	p.R781C	Het
P119	NM_001267619	*ARPP21*	c.1863C>G	p.P621P	Het
P120	NM_004429	*EFNB1*	c.406+3G>T	p.?	Het
P121	NM_021980	*OPTN*	c.160C>G	p.L54V	Het
P121	NM_001164375	*C10orf105*	c.328G>A	p.V110I	Het
P121	NM_002517	*NPAS1*	c.1167C>T	p.S389S	Het
P121	NM_001664	*RHOA*	c.156+2_156+3insA	p.?	Het

P121	NM_207411	*XKR5*	c.19G>A	p.G7R	Het
P123	NM_006977	*ZBTB25*	c.891C>G	p.L297=	Het
P123	NM_004036	*ADCY3*	c.894C>T	p.D298=	Het
P123	NM_004612	*TGFBR1*	c.944A>G	p.H315R	Het
P125	NM_004958	*MTOR*	c.1352A>G	p.Y451C	Het
P126	NM_144666	*DNHD1*	c.11402T>C	p.M3801T	Het
P126	NM_001136501	*ZNF844*	c.279C>T	p.N93=	Het
P127	NM_003482	*KMT2D*	c.6109+3A>G	p.?	Het
P127	NM_020655	*JPH3*	c.508G>A	p.E170K	Het
P127	NM_001293189	*OBP2A*	c.443C>G	p.P148R	Het
P127	NM_001001888	*VCX3B*	c.118A>G	p.K40E	Het
P128	NM_000141	*FGFR2*	c.1040C>G	p.S347C	Het
P128	NM_006640	*SEPTIN9*	c.276G>A	p.L92=	Het
P128	NM_178148	*SLC35B2*	c.688G>T	p.V230L	Het
P130	NM_021222	*PRUNE1*	c.1226C>T	p.P409L	Het
P130	NM_003036	*SKI*	c.107C>T	p.A36V	Het
P130	NM_003036	*SKI*	c.374A>T	p.Q125L	Het
P130	NM_006122	*MAN2A2*	c.1496G>A	p.R499Q	Het
P131	NM_006618	*KDM5B*	c.3803A>G	p.Q1268R	Het
P131	NM_000141	*FGFR2*	c.410C>G	p.S252W	Het
P131	NM_016615	*SLC6A13*	c.120dup	p.F41Vfs*57	Het
P136	NM_000141	*FGFR2*	c.1025G>C	p.C342S	Het
P136	NM_016113	*TRPV2*	c.601G>A	p.G201S	Het
P136	NM_005408	*CCL13*	c.88G>A	p.V30I	Het
P137	NM_006473	*TAF6L*	c.1614C>T	p.G538=	Het
P138	NM_016204	*GDF2*	c.24G>A	p.V8=	Het
P138	NM_018149	*SMG8*	c.2431G>A	p.D811N	Het
P140	NM_198839	*ACACA*	c.94G>A	p.E32K	Het
P140	NM_025185	*TANC2*	c.4234G>A	p.E1412K	Het
P141	NM_021205	*RHOU*	c.50C>T	p.P17L	Het
P141	NM_001256720	*CLEC19A*	c.173A>G	p.N58S	Het
P141	NM_001145011	*C16orf96*	c.1863A>G	p.A621=	Het

Abbreviation: HGVS, Human Genome Variation Society; Het, heterozygote

Supplemental Table 3. List of likely pathogenic or pathogenic variants associated with craniosynostosis identified in this study.

Sample	Transcript	Gene	HGVS coding	HGVS protein	GT	Origin	OMIM	ACMG criteria
P005	NM_006494	*ERF*	c.257G>A	p.R86H	Het	Pat	Craniosynostosis 4, AD (#600775)	PM1, PM2, PM5, PP3, PP4 (LP)
P007	NM_000142	*FGFR3*	c.1138G>A	p.G380R	Het	NA	Muenke syndrome, AD (#602849)	PS1, PS3, PM1, PM2, PP3, PP5 (P)
P023	NM_000478	*ALPL*	c.668G>A	p.R223Q	Het	Mat	Odontohypophosphatasia, AR, AD (#146300)	PM1, PM2, PM5, PP3, PP5 (LP)
P024	NM_001042492	*NF1*	c.3103A>G	p.M1035V	Het	De Novo	Watson syndrome, AD (#193520)	PS2, PM1, PM2, PM5 (P)
P025	NM_000090	*COL3A1*	c.1282C>T	p.R428X	Het	NA	Ehlers-Danlos syndrome, vascular type, AD (#130050)	PVS1, PM2, PP3, PP5 (P)
P028	NM_001042681	*RERE*	c.1978G>A	p.E660K	Het	De Novo	Neurodevelopmental disorder with or without anomalies of the brain, eye, or heart, AD (#616975)	PS2, PM2, PP3 (LP)
P028	NM_005585	*SMAD6*	c.1106del	p.G369Afs*170	Het	Pat	{Craniosynostosis 7, susceptibility to}, AD (#617439)	PVS1, PM2 (LP)
P036	NM_053002	*MED12L*	c.5854C>T	p.Q1952X	Het	NA	Nizon-Isidor syndrome, AD (#618872)	PVS1, PM2 (LP)
P038	NM_001852	*COL9A2*	c.1298G>C	p.G433A	Het	De Novo	Epiphyseal dysplasia, multiple, 2, AD (#600204)	PS2, PM2, PP3 (LP)
P042	NM_020791	*TAOK1*	c.2545-1_2545insT	p.I849Yfs*2	Het	De Novo	Developmental delay with or without intellectual impairment or behavioral abnormalities, AD (#619575)	PVS1, PS2, PM2, PP3 (P)
P050	NM_000474	*TWIST1*	c.395G>C	p.R132P	Het	NA	Craniosynostosis 1, AD (#123100)	PM1, PM2, PM5, PP3, PP5 (LP)
P052	NM_207037	*TCF12*	c.1774C>T	p.P592S	Het	NA	Craniosynostosis 3, AD (#615314)	PM1, PM2, PP3, PP4 (LP)
P052	NM_170606	*KMT2C*	c.7443dup	p.F2482Ifs*7	Het	NA	Kleefstra syndrome 2, AD (#617768)	PVS1, PM2 (LP)
P058	NM_207037	*TCF12*	c.627del	p.S210Vfs*35	Het	De Novo	Craniosynostosis 3, AD (#615314)	PVS1, PS2, PM2, PP3 (P)
P068	NM_207037	*TCF12*	c.1468-7A>G	p.?	Het	NA	Craniosynostosis 3, AD (#615314)	PS2, PM2, PP3 (LP)
P069	NM_001913	*CUX1*	c.1450+1G>A	p.?	Het	NA	Global developmental delay with or without impaired intellectual development, AD (#618330)	PVS1, PM2 (LP)

P070	NM_000138	*FBN1*	c.3128A>G	p.K1043R	Het	NA	Geleophysic dysplasia 2, AD (#614185)	PM1, PM2, PP3, PP5 (LP)
P070	NM_000214	*JAG1*	c.1569+1G>A	p.?	Het	NA	Alagille syndrome 1, AD (#118450)	PVS1, PM2 (LP)
P071	NM_003482	*KMT2D*	c.15461G>A	p.R5154Q	Het	NA	Kabuki syndrome 1, AD (#147920)	PM1, PM2, PM6, PP3, PP5 (LP)
P074	NM_014208	*DSPP*	c.3519delT	p.D1173Efs*141	Het	Pat	Dentinogenesis imperfecta, AD (#125500)	PVS1, PM2 (LP)
P079	NM_001145856	*PACS1*	c.194del	p.D66Tfs*40	Het	NA	Schuurs-Hoeijmakers syndrome, AD (#615009)	PVS1, PM2, PP3 (LP)
P088	NM_006494	*ERF*	c.886G>A	p.G296S	Het	NA	Craniosynostosis 4, AD (#600775)	PM1, PM2, PP1, PP4 (LP)
P091	NM_012062	*DNM1L*	c.1073C>T	p.S358L	Het	De Novo	Encephalopathy, lethal, due to defective mitochondrial peroxisomal fission 1, AR, AD (#614388)	PS2, PM2 (LP)
P098	NM_006494	*ERF*	c.985_1027del	p.R329Sfs*54	Het	Mat	Craniosynostosis 4, AD (#600775)	PVS1, PM2 (LP)
P103	NM_001353345	*SETD1B*	c.444_445del	p.K148Nfs*42	Het	NA	Intellectual developmental disorder with seizures and language delay, AD (#619000)	PVS1, PM2 (LP)
P112	NM_002547	*OPHN1*	c.1364del	p.N455Ifs*7	Hemi	Mat	Intellectual developmental disorder, X-linked syndromic, Billuart type, XR (#300486)	PVS1, PM2 (LP)
P114	NM_000474	*TWIST1*	c.466A>G	p.I156V	Het	NA	Craniosynostosis 1, AD (#123100)	PM1, PM2, PM5, PP3, PP5 (P)
P115	NM_148898	*FOXP2*	c.682C>T	p.Q228X	Het	NA	Speech-language disorder-1, AD (#602081)	PVS1, PM2 (LP)
P116	NM_032957	*RTEL1*	c.919+5G>A	p.?	Het	De Novo	Dyskeratosis congenita, autosomal dominant 4, AD (#615190)	PS2, PM2, PP3 (LP)
P118	NM_207037	*TCF12*	c.191delC	p.S64Ffs*11	Het	NA	Craniosynostosis 3, AD (#615314)	PVS1, PM2 (LP)
P120	NM_004429	*EFNB1*	c.406+3G>T	p.?	Het	De Novo	Craniofrontonasal dysplasia, XD (#304110)	PS2, PM2, PP3, PP5 (LP)
P121	NM_006494	*ERF*	c.248G>A	p.R83Q	Het	Mat	Craniosynostosis 4, AD (#600775)	PM1, PM2, PP3, PP5 (LP)
P123	NM_004612	*TGFBR1*	c.944A>G	p.H315R	Het	De Novo	Loeys-Dietz syndrome 1, AD (#609192)	PS2, PM1, PM2, PM5, PP3, PP5 (P)
P125	NM_004958	*MTOR*	c.1352A>G	p.Y451C	Het	De Novo	Smith-Kingsmore syndrome, AD (#616638)	PS2, PM2 (LP)

P126	NM_207037	*TCF12*	c.1917_1920del	p.K640Sfs*14	Het	Pat	Craniosynostosis 3, AD (#615314)	PVS1, PM2 (LP)
P127	NM_003482	*KMT2D*	c.6109+3A>G	p.?	Het	De Novo	Kabuki syndrome 1, AD (#147920)	PS2, PM2, PP3 (LP)
P128	NM_000141	*FGFR2*	c.1040C>G	p.S347C	Het	De Novo	Crouzon syndrome, AD (#123500)	PS2, PM1, PM2, PP3, PP5 (P)
P130	NM_003036	*SKI*	c.107C>T	p.A36V	Het	De Novo	Shprintzen-Goldberg syndrome, AD (#182212)	PS2, PM1, PM2, PP3, PP5 (P)
P130	NM_003036	*SKI*	c.374A>T	p.Q125L	Het	De Novo	Shprintzen-Goldberg syndrome, AD (#182212)	PS2, PM1, PM2, PP3 (P)
P131	NM_000141	*FGFR2*	c.755C>G	p.S252W	Het	De Novo	Crouzon syndrome, AD (#123500)	PS2, PM1, PM2, PP3 (P)
P135	NM_197968	*ZMYM2*	c.1868C>A	p.S623X	Het	NA	Neurodevelopmental-craniofacial syndrome with variable renal and cardiac abnormalities, AD (#619522)	PVS1, PM2 (LP)
P136	NM_000141	*FGFR2*	c.1025G>C	p.C342S	Het	De Novo	Crouzon syndrome, AD (#123500)	PS2, PM1, PM2, PP3, PP5 (P)
P138	NM_001142784	*IL11RA*	c.C886T	p.R296W	Het	Pat	Craniosynostosis and dental anomalies, AR (#614188)	PM1, PM2, PM3, PP3, PP5 (LP)
			c.811-2A>G	p.?	Het	Mat		PVS1, PM2, PP3 (LP)
P139	NM_000276	*OCRL*	c.1841G>A	p.W614X	Hemi	NA	Dent disease 2, XR (#300555)	PVS1, PM2 (LP)
P140	NM_025185	*TANC2*	c.4234G>A	p.E1412K	Het	De Novo	Intellectual developmental disorder with autistic features and language delay, with or without seizures, AD (#618906)	PS2, PM2, PP3 (LP)

Abbreviation: HGVS, Human Genome Variation Society; GT, genotype; Het, heterozygote; Hemi, hemizygote; NA, not available; MOI, mode of inheritance; AD, autosomal dominant; AR, autosomal recessive; XD, X-linked dominant; XR, X-linked recessive;

Supplemental Table 4. Case-specific digenic pairs with two unrelated cases identified in this study

Proband	Sex	CRS	Gene A (Transcript)	AA change	Origin	Gene B (Transcript)	AA change	Origin	P/LP variants in CRS or NDD genes
P051	male	S	*IL6ST* (NM_002184)	p.S248F	NA	*TRPS1* (NM_014112)	p.Q181R	NA	No
P093	female	L		p.N360S	Pat		p.R814L	Mat	No
P052	female	C	*LRP1* (NM_002332)	p.R2440W	NA	*PTPRZ1* (NM_002851)	p.F827L	NA	*TCF12*
P061	male	L		p.S1149L	Pat		p.E1336G	Mat	No
P028	male	C	*SLC4A2* (NM_003040)	p.R95H	Pat	*NELL2* (NM_001145108)	p.R122W	Mat	*RERE*
P132	female	C		p.V1155I	NA		p.A156T	NA	No
P032	female	S	*PTPN12* (NM_002835)	p.S684L	Pat	*TSC2* (NM_000548)	p.K1689R	Mat	No
P071	male	S		p.L336M	NA		p.S1383I	NA	*KMT2D*
P110	male	S	*COL12A1* (NM_004370)	p.V1862A	NA	*CACNA1A* (NM_001127222)	p.R2195Q	NA	No
P121	male	S, L		p.A2760V	Pat		p.R480C	Mat	*ERF*
P049	female	S	*BMP4* (NM_001202)	p.H251Y	NA	*FAT3* (NM_001367949)	p.Y339C	NA	No
P131	male	C		p.R162Q	Pat		p.R433W	Mat	*FGFR2*

Abbreviation: AA, amino acids; P, pathogenic; LP, likely pathogenic; CRS, cranial suture involvement; S, sagittal; M, metopic; C, coronal; L, lambdoidal; Pat, paternally inherited; Mat, maternally inherited; NA, not available;

Supplemental Table 5. Pathogenicity prediction of digenic combinations using the ORVAL.

**Gene A**	**Gene B**	**Gene A allele (coordinate)**	**Gene B allele (coordinate)**	**VarCoPP score**	**Predicted class**	**Confidence zone**
TRPS1	*IL6ST*	8:116599487:C:A	5:55252041:T:C	0.9625	Disease-causing	99.9%-zone
NUP188	*IL6ST*	9:131765210:G:A	5:55252041:T:C	0.935	Disease-causing	99.9%-zone
IL6ST	*SMG6*	5:55252041:T:C	17:1989098:T:G	0.895	Disease-causing	99.9%-zone
NUP188	*SMG6*	9:131765210:G:A	17:1989098:T:G	0.8925	Disease-causing	99.9%-zone
TRPS1	*SMG6*	8:116599487:C:A	17:1989098:T:G	0.88	Disease-causing	99.9%-zone
MAP3K4	*IL6ST*	6:161519441:G:A	5:55252041:T:C	0.875	Disease-causing	99%-zone
MAP3K4	*SMG6*	6:161519441:G:A	17:1989098:T:G	0.8275	Disease-causing	99%-zone
MAP3K4	*TRPS1*	6:161519441:G:A	8:116599487:C:A	0.7675	Disease-causing	
NUP188	*TRPS1*	9:131765210:G:A	8:116599487:C:A	0.6975	Disease-causing	
IL6ST	*HMGXB3*	5:55252041:T:C	5:149406325:G:A	0.62	Disease-causing	
SMG6	*HMGXB3*	17:1989098:T:G	5:149406325:G:A	0.475	Neutral	
TRPS1	*HMGXB3*	8:116599487:C:A	5:149406325:G:A	0.4575	Neutral	
NUP188	*HMGXB3*	9:131765210:G:A	5:149406325:G:A	0.2375	Neutral	
MAP3K4	*HMGXB3*	6:161519441:G:A	5:149406325:G:A	0.195	Neutral	
MAP3K4	*NUP188*	6:161519441:G:A	9:131765210:G:A	0.1875	Neutral	

This table provides the pathogenicity predictions for digenic combinations using ORVAL in proband P093. Rare variants were filtered according to the method described in Figure 2, and the pathogenicity of each digenic combination was predicted individually. ORVAL utilizes gene-gene interaction networks and the pathogenicity of each variant combination to predict the pathogenicity of digenic combinations. Digenic combinations with disease-causing potential, exhibiting segregated alleles in the 99.9% zone, were further evaluated in the validation cohort to identify additional cases harboring these combinations.

Supplemental Table 6. The list of reagents.

	REAGENT or RESOURCE	SOURCE	IDENTIFIER
**Antibody**			
	AKT (pan)	CST	Cat# 9272
	Aldolase	GeneTex	Cat# GTX101408
	ERK1/2	CST	Cat# 9102
	IL6ST	CST	Cat# 3732
	Myc-Tag (9B11)	CST	Cat# 2276
	Phospho-AKT (Ser473)	CST	Cat# 9271
	Phospho-ERK1/2 (Thr202/Tyr204)	CST	Cat# 9106
	Phospho-STAT3 (Tyr705)	CST	Cat# 9131
	STAT3 (79D7)	CST	Cat# 4904
	TRPS1	CST	Cat# 17936
	β-actin	GeneTex	Cat# GTX109639
**Bacterial and virus**			
	DH5α competent cell	RBC	Cat# RH617
**Chemicals, peptides, and recombinant protein**			
	DL-dithiothreitol	Sigma	Cat# 43815
	DMEM	Gibco	Cat# 11995-065
	DNase I	BioLabs	Cat# M0303S
	D-PBS	Welgene	Cat# LB001-02
	Fetal bovine serum	Gibco	Cat# 26140-079
	Halt Phosphatase Inhibitor Cocktail	Thermo scientific	Cat# 78426
	L-Ascorbic acid	Sigma	Cat# A4544-25G
	Lipofectamine RNAiMAX Reagent	Invitrogen	Cat# 13778-100
	Opti-MEM	Gibco	Cat# 31985-070
	Penicillin/Streptomycin	Gibco	Cat# 15140-122
	Purelink Rnase A	Invitrogen	Cat# 12091021
	RANKL	PeproTech	Cat# 315-11
	Recombinant Human IL-11	PeproTech	Cat# P20809
	RIPA lysis buffer	Thermo Scientific	Cat# 89900
	TransIT-LT1 Transfection Reagent	Mirus Bio	Cat# MIR2300
	Tri-RNA Reagent	FAVORGEN	Cat# FATRR001
	αMEM without L-ascorbic acid	Welgene	Cat# LM008-53
	aMEM	Welgene	Cat# LM008-01
	β-Glycerophosphate disodium salt hydrate	Sigma	Cat# G9422-50G
**Critical commercial assays**			
	AccuPrep Universal RNA extraction kit	Bioneer	Cat# K-3140
	ALP assay	Abcam	Cat# ab83369
	ALP staining kit	Wako	Cat# 204-67001
	Dual-Luciferase Reporter Assay system	Promega	Cat# E1910
	Quick-DNA miniprep plus kit	Zymo	Cat# D4068
	TRAP staining kit	Wako	Cat# 204-67001
	RNA to DNA EcoDry Premix	TaKaRa	Cat# 639549
	TaKaRa BCA Protein Assay kit	TaKaRa	Cat# T9300A
	TIANprep Mini Plasmid Kit	TIANGEN	Cat# 4992420
**Experimental models: Cell line**			
	HEK293	ATCC	CRL-3216
	HepG2	ATCC	HB-8065
	MC3T3-E1	ATCC	Cat# CRL-2593

	RAW264.7	ATCC	Cat# TIB-71
**Recombinant DNA & CDS clone**			
	IL11RA	OriGene	Cat# RC200654
	IL6ST	OriGene	Cat# RC215123
	p6OSE2-Luciferase vector	N/A	
	pCMV6-Entry	OriGene	Cat# PS100001
	pGL4.47[luc2P/SIE/Hygro]	Promega	Cat# E4041
	pGL4.70[hRluc]	Promega	Cat# E6881
	RUNX2	N/A	
	TRPS1	OriGene	Cat# RC215856

Supplemental Table 7. Sequence information.

Name	F/R	Sequence
**RT-PCR**		
GAPDH	F	5'-CATCACTGCCACCCAGAAGACTG-3'
	R	5'-ATGCCAGTGAGCTTCCCGTTCAG-3'
IL6ST	F	5'-CTCTGAGTCCTTGAAGGCGTAC-3'
	R	5'-CCATTCTGGTCGTCCACAGGAA-3'
TRPS1	F	5'-CAACCGTTCTGTGCTTTCTGGC-3'
	R	5'-GTGTTGCCTTGGCAATCTGGAG-3'
IBSP	F	5'-AATGGAGACGGCGATAGTTCCG-3'
	R	5'-GGAAAGTGTGGAGTTCTCTGCC-3'
BGLAP1	F	5'-GCAATAAGGTAGTGAACAGACTCC-3'
	R	5'-CCATAGATGCGTTTGTAGGCGG-3'
Col1A1	F	5'-CCTCAGGGTATTGCTGGACAAC-3'
	R	5'-CAGAAGGACCTTGTTTGCCAGG-3'
RUNX2	F	5'-CCTGAACTCTGCACCAAGTCCT-3'
	R	5'-TCATCTGGCTCAGATAGGAGGG-3'
ALPL	F	5'-CCAGAAAGACACCTTGACTGTGG-3'
	R	5'-TCTTGTCCGTGTCGCTCACCAT-3'
CTSK	F	5'-AGCAGAACGGAGGCATTGACTC-3'
	R	5'-CCCTCTGCATTTAGCTGCCTTTG-3'
DCSTAMP	F	5'-TTTGCCGCTGTGGACTATCTGC-3'
	R	5'-GCAGAATCATGGACGACTCCTTG-3'
TRAP	F	5'-GCGACCATTGTTAGCCACATACG-3'
	R	5'-CGTTGATGTCGCACAGAGGGAT-3'
NFATc1	F	5'-GGTGCCTTTTGCGAGCAGTATC-3'
	R	5'-CGTATGGACCAGAATGTGACGG-3'
**siRNA**		
siIL6ST	F	5'-CUGCUUAUUCUGUAGUGAAdtdt-3'
	R	5'-UUCACUACAGAAUAAGCAGdtdt-3'
	F	5'-GUGCUAUCAAAUCACAGUAdtdt-3'
	R	5'-UACUGUGAUUUGAUAGCACdtdt-3'
	F	5'-CUCGAACUUCCUUCACUGUdtdt-3'
	R	5'-ACAGUGAAGGAAGUUCGAGdtdt-3'
siTRPS1	F	5'-CUCAUGUGUUUCUGAUCAUdtdt-3'
	R	5'-AUGAUCAGAAACACAUGAGdtdt-3'
	F	5'-CUGCUAAACCCAGACUCUAdtdt-3'
	R	5'-UAGAGUCUGGGUUUAGCAGdtdt-3'
	F	5'-CUGCAAUAGGUUGUCUACAdtdt-3'
	R	5'-UGUAGACAACCUAUUGCAGdtdt-3'
siIL6ST(3'-UTR)	F	5'-GAUGUUUGCACUGAAGAAAdtdt-3'
	R	5'-UUUCUUCAGUGCAAACAUCdtdt-3'
**Site-directed mutagenesis**		
TRPS1_Q168R	F	5'-ATTGGCTTGACCACTCCGTGCTTGCCCTGTTTC-3'
	R	5'-GAAACAGGGCAAGCACGGAGTGGTCAAGCCAAT-3'
TRPS1_R801L	F	5'-TGACGGACTCCCCAGCAGGATGTCTGC-3'
	R	5'-GCAGACATCCTGCTGGGGAGTCCGTCA-3'
IL6ST_N360S	F	5'-TCCAAGATTTTTCCACTGGCTTCAAAAGGAGGCAATGTC-3'
	R	5'-GACATTGCCTCCTTTTGAAGCCAGTGGAAAAATCTTGGA-3'
IL6ST_S580F	F	5'-AATGTGTCACTAGTCAAAGAGAACAATGTATATTCTGTGTGGG-3'

	R	5'-CCCACACAGAATATACATTGTTCTCTTTGACTAGTGACACATT-3'
**Patient genotyping**		
TRPS1_Q181R	F	5'-TCAAGATATGGCCTGCACCCCCTC-3'
	R	5'-TTTGGATTTATTCAGTCTTACACCCCCA-3'
TRPS1_R814L	F	5'-CCACCATCAAAGAGGAGCCCAAAAT-3'
	R	5'-CACAGCCAAGCCATAAATAGGTCGC-3'
IL6ST_N360S	F	5'-GCGATCATTTGTGAGATTTACTGTCAGTTTTGTG-3'
	R	5'-GACATAATGGCATGATTTGTGAATATGAAG-3'
IL6ST_S580F	F	5'-TTCTGGACCATCCTTCCCACCTTC-3'
	R	5'-CTTCCAGCTGTGAATGTGGATTCTTCCC-3'
TRPS1_Q181R	F	5'-TCAAGATATGGCCTGCACCCCCTC-3'
	R	5'-TTTGGATTTATTCAGTCTTACACCCCCA-3'
**Patient genotyping (Sanger sequencing)**		
P024_NF1	F	5'-CACTGATACTGGTAGTAATTGATAAAATAACTGG-3'
	R	5'-TTCATTGGACATATTAAGATTTACAAGACC-3'
P028_RERE	F	5'-TGGAACTCAGAATGAGGGAGCAAAGTGT-3'
	R	5'-GCTCCGGGACCACAGGTCGTG-3'
P038_Col9A2	F	5'-CAAGGCTTGCCAGGCGTCAAA-3'
	R	5'-ACAAGGAGCAGCGGTCACGAAGC-3'
P042_TAOK1	F	5'-AATCTGCAGTTGTACCCCCGAATATATAA-3'
	R	5'-TACCCCTTGCATTGGCCCTGAA-3'
P058_TCF12	F	5'-ACTGTCATACCAAGATGCATTACAGAGAT-3'
	R	5'-GCAGTCAGCAATAAACACCACTGGAA-3'
P116_RTEL1	F	5'-GCCACACAGTCATGTTTGGACCT-3'
	R	5'-GTGCCAGCCTGAATAGATGGTGCCC-3'
P120_EFNB1	F	5'-GTGTTGGTCACCTGCAATAGGCCAGAG-3'
	R	5'-CTAAGAGGAGAACATGCCAGTCTTCAAAGG-3'
P123_TGFBR1	F	5'-TGTGAAGGAAATACAGACTTAAGGTGGCA-3'
	R	5'-AGGCTGGCCATGAACTCCTGAGATC-3'
P125_MTOR	F	5'-TCTCCAAGATACCATGAACCATGTCCTAA-3'
	R	5'-ATGATGCAAAAAATGGGCGTAAGCTC-3'
P127_KMT2D	F	5'-GGTGAGGGCGACGGACTCTCCT-3'
	R	5'-GCTTTGTCAGCTGCTGGAACCTTTC-3'
P128_FGFR2	F	5'-CTTTTCTTTTGCTTCCCTTGTTTTCTAGG-3'
	R	5'-GAAGCTGTGTTAATTTTATAGCAGTCAACCA-3'
P130_SKI	F	5'-CTGCAGAAGACGCTGGAGCAGTTCC-3'
	R	5'-CATGACTTTGAGGATCTCCAGCTGGTC-3'
P131_FGFR2	F	5'-ATGGGGCCACAGTGTTATTTCAAAGGT-3'
	R	5'-AATCAAAGAACCTGTGGCCAAACCC-3'
P136_FGFR2	F	5'-CTTTTCTTTTGCTTCCCTTGTTTTCTAGG-3'
	R	5'-GAAGCTGTGTTAATTTTATAGCAGTCAACCA-3'
P140_TANC2	F	5'-GCAGCAGCCTTAGAGGACCTGAACGA-3'
	R	5'-GGGCAGGTGAGGTGGACTGATAGGTCT-3'
