# Supplementary material for: Digenic impairments of haploinsufficient genes in patients with craniosynostosis
Source: JCI Insight. 2025 Feb 24;10(4):e176985. doi: 10.1172/jci.insight.176985 (PMC11949007; doi:10.1172/jci.insight.176985)

Unedited Western blot image for Figure 3.F

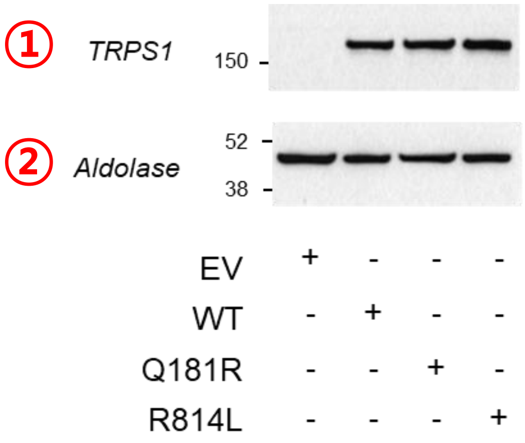

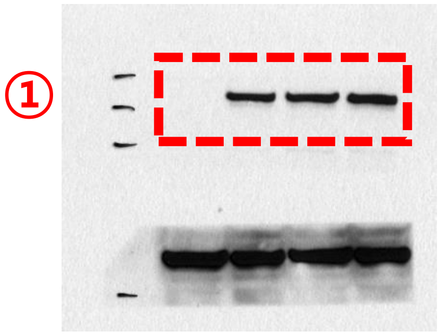

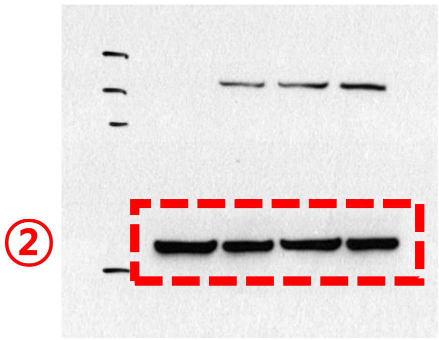

Unedited Western blot image for Figure 3.H

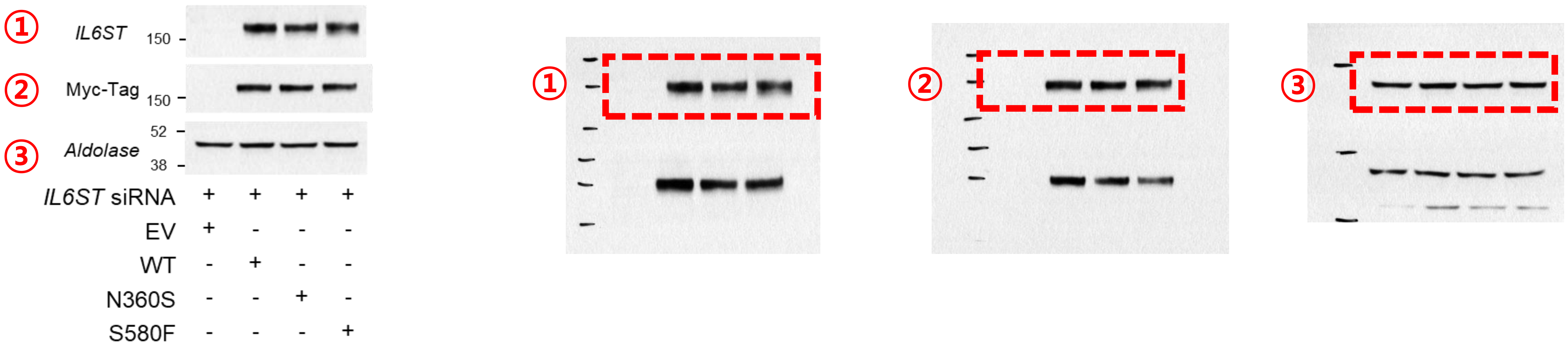

Unedited Western blot image for Figure 4.F

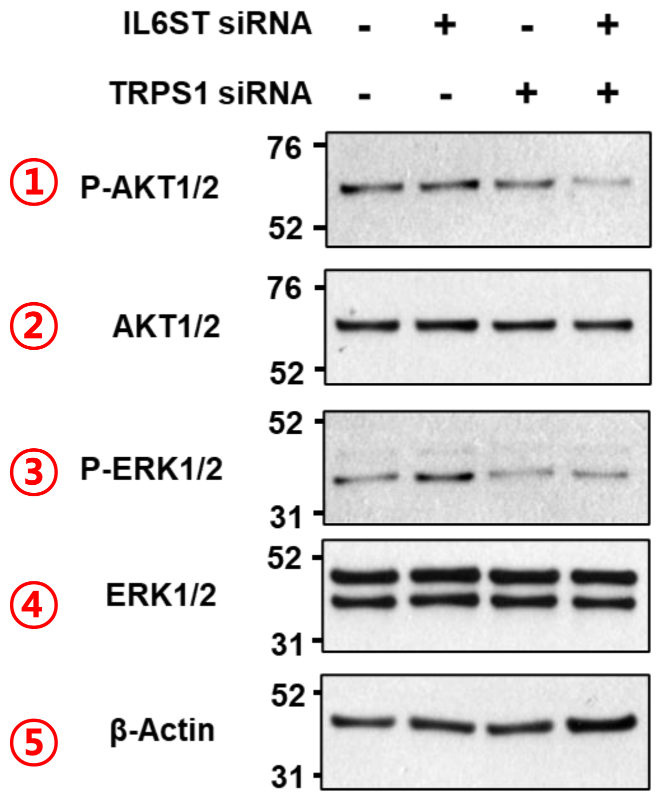

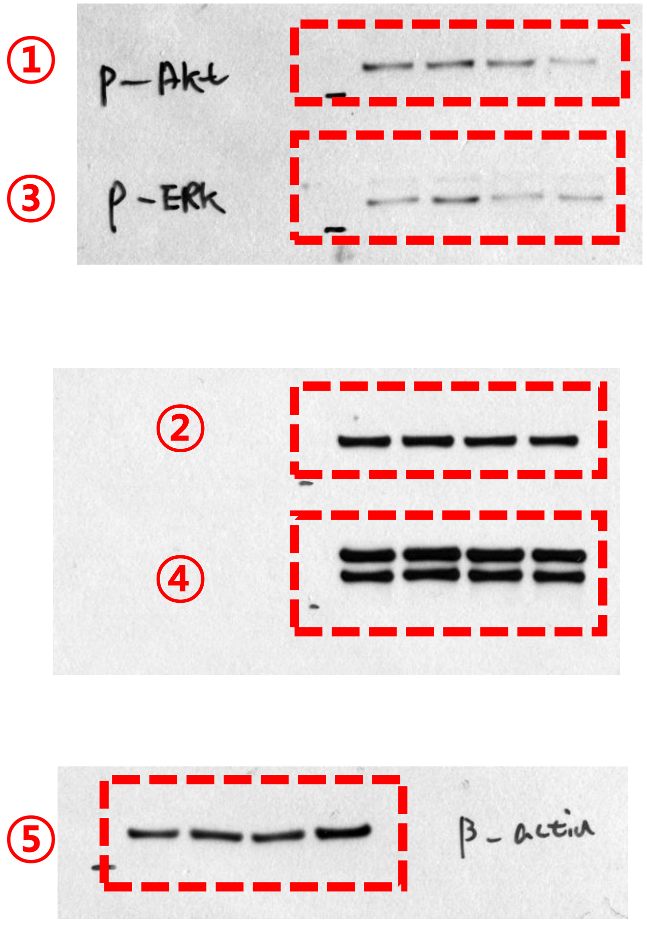

Unedited Western blot image for Supplemental Figure 7.C

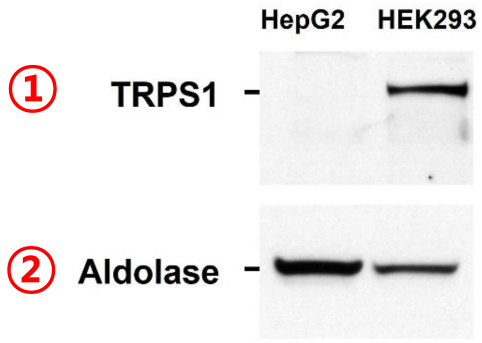

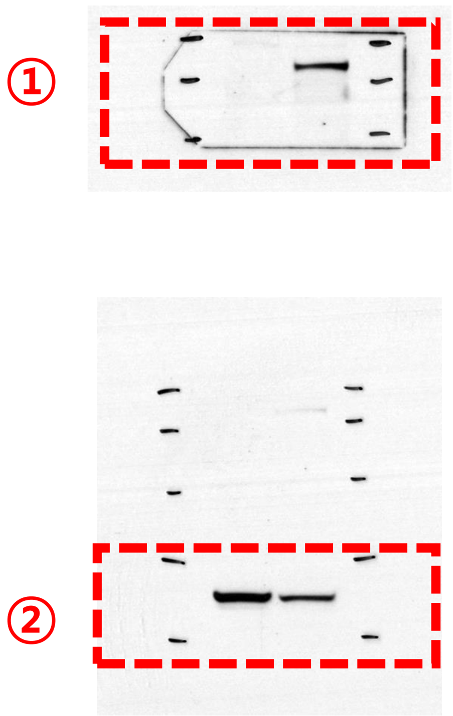

Unedited Western blot image for Supplemental Figure 7.D

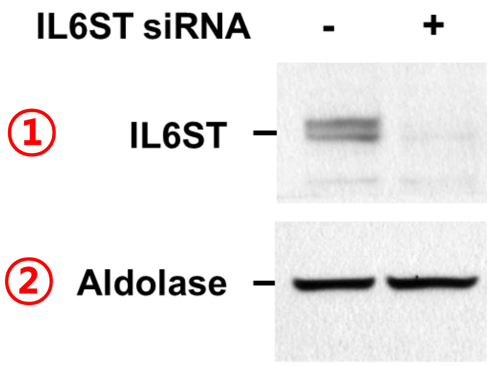

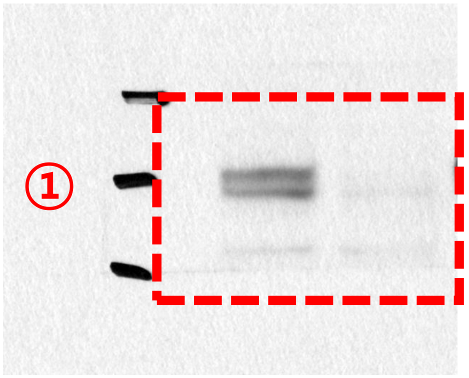

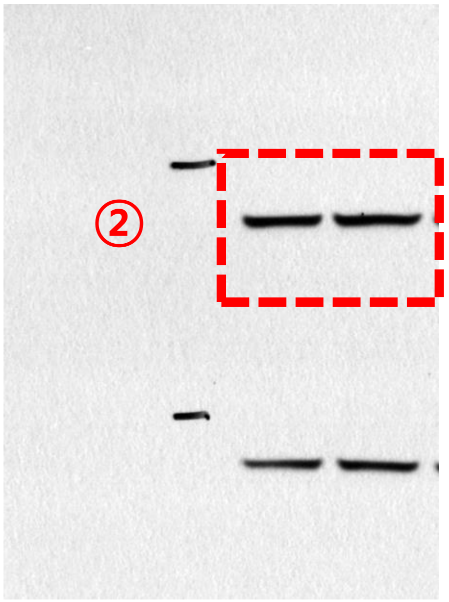

Supplement: Unedited blot and gel images [file jciinsight-10-176985-s043.pdf]
